# Single-trial detection of auditory cues from the rat brain using memristors

**DOI:** 10.1126/sciadv.adp7613

**Published:** 2024-09-04

**Authors:** Caterina Sbandati, Spyros Stathopoulos, Patrick Foster, Noam D. Peer, Cristian Sestito, Alex Serb, Stefano Vassanelli, Dana Cohen, Themis Prodromakis

**Affiliations:** ^1^ Centre for Electronics Frontiers, Institute for Integrated Micro and Nano Systems, School of Engineering, The University of Edinburgh, Edinburgh, UK.; ^2^ The Gonda Brain Research Center, Bar-Ilan University, Ramat-Gan 52900, Israel.; ^3^ Padua Neuroscience Center, University of Padua, via Orus 2/B, 35131 Padua, Italy.

## Abstract

Implantable devices hold the potential to address conditions currently lacking effective treatments, such as drug-resistant neural impairments and prosthetic control. Medical devices need to be biologically compatible while providing enhanced performance metrics of low-power consumption, high accuracy, small size, and minimal latency to enable ongoing intervention in brain function. Here, we demonstrate a memristor-based processing system for single-trial detection of behaviorally meaningful brain signals within a timeframe that supports real-time closed-loop intervention. We record neural activity from the reward center of the brain, the ventral tegmental area, in rats trained to associate a musical tone with a reward, and we use the memristors built-in thresholding properties to detect nontrivial biomarkers in local field potentials. This approach yields consistent and accurate detection of biomarkers >98% while maintaining power consumption as low as 4.14 nanowatt per channel. The efficacy of our system’s capabilities to process real-time in vivo neural data paves the way for low-power chronic neural activity monitoring and biomedical implants.

## Teaser

Nano-watt memristor-based system identifies in vivo local field potential patterns from reward-based neural pathways.

## INTRODUCTION

Highly capable signal processing microchips are critically important for bio-implant systems to enable ongoing activity-dependent intervention and personalized, self-adjusting treatments (e.g., by electrical neuromodulation) (*1*–*4*). A common theme across these areas is the constant race for lower power (*5*, *6*), higher accuracy (*7*), lower latency (*8*), and smaller physical footprint (*9*). Improvements in these basic performance metrics will enable smarter prostheses that perform more powerful computations, interact with biology in a closed-loop system, and operate with smaller batteries simultaneously (*10*).

Biosignal recording capability is crucial for advanced biomedical devices that require inputs from biology to function properly. Generally, we can record the electrical activity of the brain at various levels, ranging from scalp electroencephalograms (EEGs) to intracortical recordings acquired with implantable electrodes. The latter is more invasive and operates at lower spatial coverage but higher resolution, from whole-brain coverage at the level of brain rhythms to a few cubic millimeters at single-cell resolution. Each modality has a unique trade-off of features and challenges (*11*, *12*).

In this article, we focus on high-resolution intracortical electrophysiology, recorded by small size electrodes in the brain. The recorded current is a superposition of multiple ionic processes, and includes features ranging from fast action potentials (APs) to slower fluctuations, e.g., local field potentials (LFPs) (*13*). APs, or spikes, are extracted by high-pass filtering the raw recording, typically above 300 Hz. Distinctive AP shapes (*14*) allow the extraction of single neurons’ firing rates from the activity of multiple neurons recorded by single electrode using spike sorting techniques to yield many single-unit responses (*15*, *16*). However, stable, long-term recordings of individual neurons present issues with stability of the electrode positioning, and implementing spike sorting on-chip is power-intensive. A recent study in the field indicates that spike sorting is not an essential data preprocessing step for analyses focused on neural population activity, demonstrating the effectiveness of the multi-unit threshold crossing method (*17*). In a similar vein, other lower-frequency signals such as LFPs (<200 Hz) (*18*) present further alternatives for long-term implants. These lower-frequency potentials have a different set of characteristics and are gaining in popularity for behavioral monitoring and neural interfaces (*19*–*22*). LFPs are believed to aggregate synaptic inputs from both local and distant areas, which translate into a broader spatial resolution than APs (*23*), and on top of their robust temporal stability, they require reduced frequency to sample them. Both APs and LFPs processing present key engineering challenges, including maintaining low power and achieving reliably accurate detection of our signal of interest.

These challenges have incentivized solutions such as miniaturization, reducing computation energy (*24*), and on-node computing (i.e., computing at the extreme edge), which minimizes data transfer power (*25*). In this context, memristive devices (*26*) are emerging as a crucial bio-electronic interface that merges biosensing with computation (*27*). Known for their ability to emulate synaptic processing (*28*, *29*), memristive devices have been proposed at an early concept level as physical processors for analog signals and hybrid analog/digital computing architectures.

Notably, the memristive integrating sensor (MIS) platform concept (*30*) has yielded successful strategies for using memristors in the detection and sorting of spikes, leveraging the intrinsic threshold switching capabilities of the device to identify APs. This approach has enabled the direct application of signals to devices without prior digitalization, showcasing the platform’s innovative use of memristive technology in neural signal processing.

Furthermore, recent advancements have seen memristors being used to process LFPs (*31*, *32*), for instance converting temporally evolving signals into spatial energy maps. These maps are analyzed by a linear classifier, specifically for detecting epileptic seizures. In this dynamic landscape, we believe that the straightforward and efficient MIS concept can be extended to LFP neural data. This new type of signal offers undoubted benefits for chronic monitoring of neural state due to its improved temporal stability. Moreover, the lower bandwidth that characterizes it makes shifting towards an analog/mixed-signal solution more competitive (*33*, *34*).

In our work, we adopted the MIS concept and proposed a processing strategy for detecting behavioral events in LFPs of freely moving rats. Specifically, an awake and freely moving rat, following conditioning training, was stimulated with an auditory cue that it had learned to associate with a reward. A memristor-based system processes the neural activity in the reward-memory region of its brain with the aim of identifying specific patterns induced by the auditory cues. In this single-trial processing approach, we bypass traditional averaging steps and successfully isolate the signal of interest directly from other neural activity using data from just one instance at a time. This processing platform not only preserves the energy efficiency inherent to the MIS concept but also offers very high detection performance, facilitating the extraction of relevant behavioral patterns from neural signals in an in vivo setting.

This paper presents key contributions along three fundamental axes:

1) Adapting the MIS system concept for LFPs.

2) Showing a series of fundamental results within a behavioral setting, using single-trial in vivo data from a memory-related neural circuit site of a freely moving rat.

3) Demonstrating the accuracy performance for projected system power.

These advancements were enabled by a strategic approach that combines selecting LFPs as the neural input and using memristor devices for feature extraction. The combination not only optimizes our processing techniques but also simplifies our classification strategy, showcasing the potential of our method in neural signal processing.

## RESULTS

### Experimental framework: Analog signal chain design

**Fig. 1.**
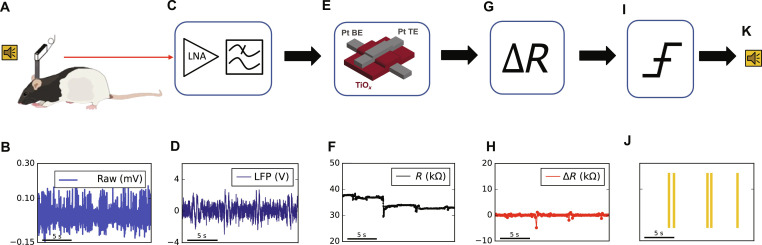
Signal processing flow in auditory cue detection using memristor devices. (**A** and **B**) We recorded neuronal activity with a microwire rendering extracellular activity of the VTA; the microwire is fully described in (*35*). (**C** and **D**) Signal from one channel is amplified and filtered via a low noise amplifier (LNA), resulting in LFP activity. (**E** and **F**) Signal is then applied to a memristive device, eliciting changes in its resistive state (*38*). (**G** and **H**) System captures these resistive changes in the memristor. (**I** and **J**) Appropriate thresholds are set to flag each detected auditory cue. (**K**) Last, the system classifies the detected signals based on the set threshold.

An experimental framework was established with the objective of detecting events influencing animal behavior through the analysis of neural activity in rats, as depicted in Fig. 1.

In the experiment, the neural activity of a freely moving rat was monitored while a sound was repetitively played, as illustrated in Fig. 1A. Preceding the experiment, the rat underwent associative conditioning training (*35*) and developed responsiveness to a specific auditory cue—namely, a 7-kHz sine wave. Drawing on foundational insights from cognitive neuroscience, which maps brain areas associated with diverse behaviors, ventral tegmental area (VTA) was chosen for recording the rat’s brain activity, a recognized site for memory development (*36*). Here, 16 microwires were chronically implanted to discern real-time neural response to the auditory cues. The signal was acquired from microwires at a frequency of 40 kHz, as depicted in Fig. 1B. The acquired voltage trace was low-pass–filtered at 130 Hz to extract LFPs and amplified by a factor of approximately 10^4^ to adjust the signal to the voltage range required by the memristor (±3 V), as shown in Fig. 1C. This process yielded the waveform depicted in Fig. 1D, while a detailed description of the front-end circuit is provided in fig. S2.

Next, the amplified LFP signal was applied to a memristive device in an analog fashion through an external board (Fig. 1E) (*37*). This hardware orchestrated the alternation between two modalities: a bias phase, where the top electrode of the memristor was connected to the neural signal, and a read-out phase, where the resistance of the memristor was measured (details of the instrument and experimental set-up in figs. S3 and S5). The period for biasing and read-out was 2 ms, corresponding to a frequency of 500 Hz. The board also sampled the LFP signal while it was being applied through an integrated high impedance voltage meter. The voltage trace was subsequently used for alignment with the memristor resistive state readings for further analysis.

The memristor was responsible for encoding the presence of patterns in the LFP through resistive state variations over time; the resistive state of the memristor was read at fixed intervals (Fig. 1F). Resistance readings were then processed by a digital circuit to evaluate resistive drops (Δ*R*) (Fig. 1, G and H). The Δ*R*s served as our biomarkers for neural activity and were input into a threshold-based binary classifier (Fig. 1, I and J), which determined whether the resistance displacements generated by the LFPs were elicited by the auditory cue. The algorithm flagged each detected auditory cue, as illustrated in Fig. 1K.

### Memristor compression and encoding of LFP

To decode the relationship between musical tone and neural activity, we applied the LFP trace acquired from the VTA of a rat to a TiO*_x_*-based memristor (*38*). One channel of the 16 surgically implanted in the rat was selected for processing; the channel offered a representative and robust LFP activity. It is noteworthy that the recorded channels exhibited a substantial degree of correlation, as shown in fig. S4.

**Fig. 2.**
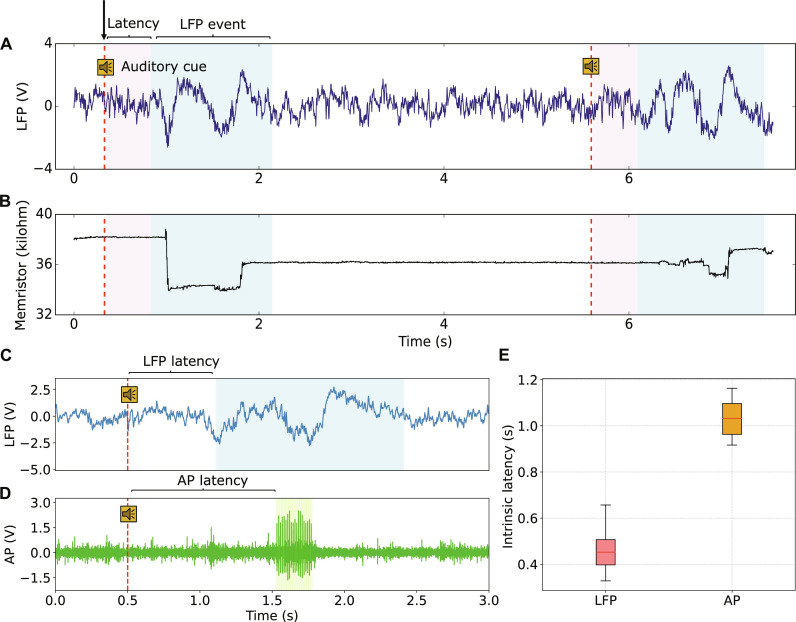
Memristor compression and encoding of LFPs. (**A**) Snippet of the amplified LFP trace. (**B**) Memristor’s resistive state acquired in real time. Red dashed lines mark instants when auditory cues were played to the rat, and light blue windows identify regions of LFP activity where the effect of the auditory cue manifests (defined here as LFP event). The LFP events occur approximately 450 ms after the auditory cue, highlighted in light pink windows. No meaningful change in memristor resistance is observed outside an event window area. (**C** and **D**) Shows a comparison of LFP and AP waveforms for the same recording channel in response to an auditory cue. Both traces were amplified. The pattern of interest in the LFP case comes before the AP, leading to a shorter neural latency. Here, the amplitude of the LFP signal has been amplified by a factor of 10^4^, ensuring that high peaks in the LFP reach the threshold voltages required for the memristor to change resistance. The AP waveform was also amplified for comparison. (**E**) Box-plot of LFP and AP intrinsic neural latency throughout the experiment.

Figure 2A showcases a segment of the LFP trace, while Fig. 2B depicts the corresponding response of the memristor to the input voltage. Here, dashed red lines pinpoint instances when the auditory cue was played to the animal. The memristor received LFP signals at a frequency of 500 Hz, coupled with read-outs taken in between episodes of neural signal stimulation. A custom-adapted daughterboard facilitated the stimulation of the memristive device with the amplified neural analog signal (refer to fig. S5 for details).

Memristive devices act as bipolar, thresholded, weighted integrators, changing their resistive state based on the applied voltage: Low voltages result in no substantial change, while high voltages cause large displacement (e.g., applying one polarity of voltage increases the resistance, and the opposite reduces it). Their change of state is driven by mechanisms such as filament creation from metal-oxide depletion (*26*) or interface barrier modulation (*39*).

Within the LFP pattern induced by the conditioning tone cue (Fig. 2A), a distinct event emerged, marked by discernible positive and negative peaks enclosed in a light blue window in the figure. Activity within these windows triggered variations in the memristor’s resistive state, as illustrated in Fig. 2B (examples of experimental traces in fig. S6). During the experiment, substantial shifts in the memristor’s resistive state coincided with LFP events indicating the potential to detect relevant LFP patterns through memristor readouts. Outside these periods, the resistive state remained stable, due to the memristor’s thresholded response. Memristors used in this experiment featured a nonvolatile response, enabling them to retain memory of suprathreshold LFP peaks even after the peaks had subsided, thus demonstrating both integrating and denoising capabilities (*40*). Here, the memristor exhibited a low resistive state (LRS) of approximately 20 kilohm and a high resistive state (HRS) of approximately 40 kilohm, with an LRS/HRS ratio of 2. However, the exact resistance values of the memristor are not crucial; our attention is primarily directed toward the change in resistance Δ*R*.

Figure 2 (C to E) presents a comparison between the characteristic responses of LFPs and APs to an auditory cue. Specifically, we note the inherent latency that marks the LFP activity (Fig. 2C) versus the AP bursts (Fig. 2D). In this case, it is evident that LFPs provide an opportunity for earlier detection of the auditory cue compared to APs. Extending our analysis to 22 response samples reinforced this finding, with LFPs consistently demonstrating a shorter delay time, as shown in Fig. 2E. This observation has substantial implications, as the reduced latency associated with LFP signals compensates for the longer latency introduced by a lower sampling frequency. APs are typically sampled at more than 12 kHz, while LFPs are sampled at ~500 Hz.

### Online detection of auditory cue

Real-time detection of auditory cues entails extracting relevant biomarkers from the memristor resistance and feeding them into a binary classification model to identify event presence. In this context, the terms biomarker and feature are used interchangeably to denote a measurable indicator of a biological state or condition. In our case, they are represented by quantities derived from changes in memristor resistance. In this section, we outline the feature extraction and detection process that takes as input the raw memristive state readouts.

**Fig. 3.**
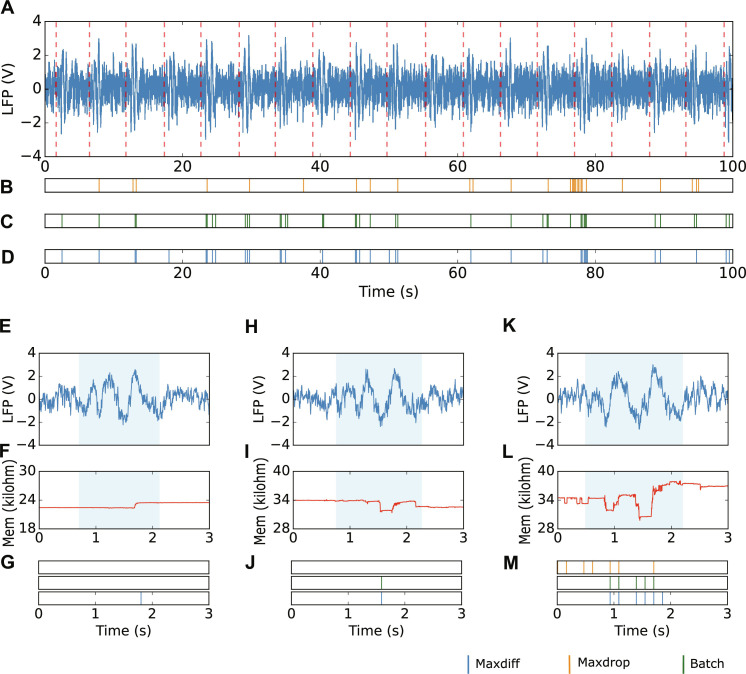
Auditory cue detection with memristive device. (**A**) LFP trace for the detection experiment. (**B** to **D**) Illustrate three different strategies to interpret the memristive device’s response respectively: maxdiff (B), maxdrop (C), and batch (D). In the rest of the article, batch approach is preferred over the others. During this experiment, the batch-size parameter *N* was set to 70; thus, each subset of values covered a time span of 140 ms. We show here a longer experiment lasting for 100 s, which includes a demonstration of event detection output using three different thresholding methods. (**E** to **G**) LFP signal exhibits relatively low peaks, resulting in a minimal memristive response. (**H** to **J**) LFP event induces conductance modulation, accurately detected by two of the three techniques. (**K** to **M**) Higher amplitude LFP rippling applies more energy to the memristive device, leading to multiple detections. Notably, the maxdiff technique displays faulty misdetection as it misinterprets the resistance instability preceding the event window.

Figure 3A presents an extended snapshot from the live experiment, displaying the rat’s neural LFP activity along with the instances when auditory cues were played. Raster plots in Fig. 3 (B to D) showcase detections carried out using three distinct methods for biomarker extraction. In all three cases, resistance readouts were processed in batches of values, with a feature being derived for each subgroup of values. This process facilitated the submission of a biomarker to the classifier, enabling outputs to be generated at a rate proportional to the batch-size *N*. Table 1 summarizes mathematical formulas for biomarker extractions. These different approaches were designed to explore effective ways of deriving meaningful resistive changes in each subset of resistive states. In particular, maxdiff aims to detect abrupt changes by identifying the largest difference between consecutive readings (*R*_curr_ and *R*_prev_) within each batch (Fig. 3B). Maxdrop focuses on the largest displacement within a batch by subtracting the highest (*R*_max_) from the lowest (*R*_min_) resistive state (Fig. 3C). Meanwhile, batch calculates the resistive change between the first (*R*_init_) and last states (*R*_end_) within the batch (Fig. 3D).

**Table 1.** Feature extraction methods.

Strategy	Formula
maxdiff	**max**(***R***_**curr**_**−*R***_**prev**_)
maxdrop	***R***_**max**_ **−*R***_**min**_
batch	|***R***_**init**_**−*R***_**end**_|

In Fig. 3 (B to D), a vertical line signifies that at the end of a specific batch, the value calculated using the corresponding formula from Table 1 satisfied the thresholding condition

$$\text{biomarker}>A∙R_{\text{init}}$$ (1)

where *R*_init_ is the value of the readout at the beginning of a batch and *A* is a scaling constant determined via a calibration and optimization method applied to each strategy from Table 1. The results presented in Fig. 3 were obtained by setting the threshold *A*, respectively: *A*_maxdiff_ = 0.03, *A*_maxdrop_ = 0.01, *A*_batch_ = 0.022. The calculation of *A* values is deferred to the next section, where an optimization strategy for the threshold is proposed.

Figure 3 (E to M) illustrate zoomed-in views of specific auditory cue events and distinctive detection performance of the three strategies. The LFP activity is colored in blue, and the resistive response of the memristor is in red, while a light blue window highlights accentuated rippling areas of the LFP. In Fig. 3 (E to G), the LFP exhibits a response characterized by smaller voltage peaks, resulting in a minimal resistive response in the memristor. This lower voltage affects the detection for maxdiff and maxdrop, as evidenced by the absence of a detection in the light blue window. Figure 3 (H to J) present another scenario where an LFP event induces resistance modulation, which enables a correct detection in two of the three techniques (maxdrop and batch). A third scenario is shown in Fig. 3 (K to M), where a higher amplitude LFP rippling applies more energy to the memristive device. Although multiple events are detected, they are acceptable if falling within the same window, as they can be grouped into a behavioral detection. However, for the maxdiff strategy, small resistance bumps preceding the event window triggered detections outside this window, counted as misdetections.

### Optimization of system performance

A comprehensive study was undertaken to fine-tune and optimize the system parameters. In particular, a systematic analysis of the three strategies proposed in Table 1 was carried out to identify the one that offers the best tradeoff in terms of detection performance, power, and computational complexity. In parallel, parameters characterizing the classification system were tuned to achieve optimal performance. Latency and power consumption analysis for the entire system are also reported and discussed.

**Fig. 4.**
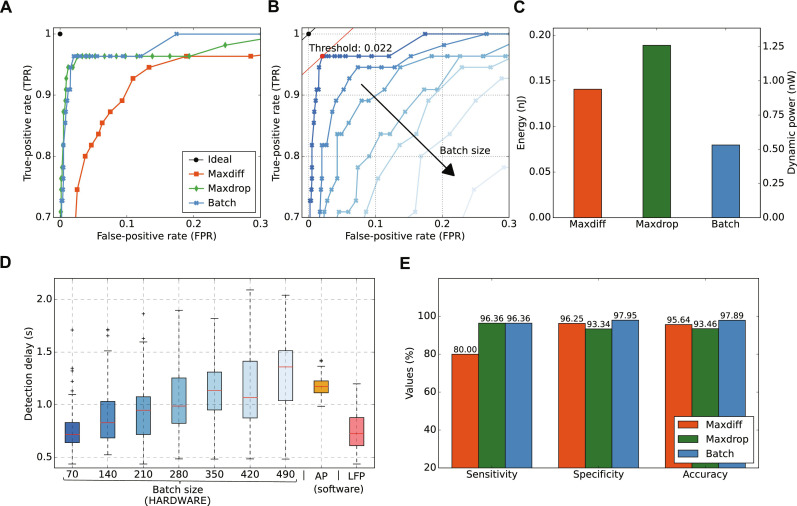
Tuning experimental parameters for optimum accuracy, latency, and computational complexity. (**A**) ROC curves for maxdiff, maxdrop, and batch post-processing strategies to identify superior detection performance. (**B**) ROC curve for a set of batch-sizes *N* = [70,140,210,280,350,420,490]. The optimal threshold value *A* = 0.022, highlighted in red, showcases the best balance between true positives and false positives. The batch strategy is investigated in further detail as it strikes a balance between accuracy and complexity; refer to figs. S7 and S8 for maxdiff and maxdrop analysis. (**C**) Energy per operation and dynamic power cost for the feature extraction stage, implemented on a field-programmable gate array (FPGA), of our system. See table S1 for details. (**D**) Detection latency, increasing with larger batch sizes, suggests that a batch size of 70 optimizes detection speed to match that of algorithmically identified LFP or AP events without requiring extensive computational resources. (**E**) At optimal setting (threshold coefficient respectively *A*_maxdiff_ = 0.03, *A*_maxdrop_ = 0.01, *A*_batch_ = 0.022, and batch-size *N* = 70), sensitivity, specificity, and accuracy for the three methods are presented.

In terms of detection performances, following established classification evaluation practices, feature extraction methods were compared using the receiver operating characteristic (ROC) curve, as shown in Fig. 4A. ROC curves, a standard method for assessing binary classifiers in clinical and bioinformatics fields (*41*), plot true-positive rate (TPR) against false-positive rate (FPR) at various threshold settings. This approach facilitated comparison of detection performances across different tests, where TPR and FPR respectively measure the system’s ability to correctly identify events and to accurately exclude nonevent (definitions in Materials and Methods). In addition, ROC curves enabled to identify an optimal threshold value for distinguishing between positive and negative states, thereby maximizing detection accuracy. In this representation, an ideal classifier would be indicated by a black dot at the point (0, 1), signifying perfect detection, while a diagonal line from (0, 0) to (1, 1) represents a random guess. Comparing ROC curves in Fig. 4A revealed that the maxdrop and batch strategies have similar levels of performance, while the maxdiff strategy’s ROC curve showed a less pronounced elbow, suggesting that it was comparatively less effective.

The batch method not only showed a promising ROC curve but also offered the simplest computational approach, an aspect that will be elaborated later in this section. Therefore, in Fig. 4B, the performance of the batch method was further investigated, examining variations of the ROC curve for different batch-size *N*, which represents the number of samples from which our biomarkers are extracted. The batch size was initialized to *N* = 70, corresponding to a window of 140 ms; this was deemed a reasonable minimum batch size as it roughly represents ^1^/_12_ of the duration of an LFP event. The figure shows comparative analysis, from dark blue corresponding to *N* = 70 up to *N* = 490 in pale blue. It emerged that the highest performance was offered by a batch size of *N* = 70, as the corresponding curve is closer to the ideal classifier. Batch-size analysis for maxdiff and maxdrop are depicted in figs. S7A and S8A.

Figure 4B also depicts the proposed strategy for identifying optimal threshold coefficient *A* (Eq. 1). In particular, the optimal *A*_batch_ value was determined using the minimal secant method. This method involved positioning a secant line at α = 45^°^ angle relative to the *x* axis and sliding it across all points on the ROC curve. As a reminder, each point on a given ROC curve represents the detection performance at a specific threshold, although the threshold values do not explicitly appear on the ROC graph. For each point, the Euclidean distance from the secant line to the line cutting the ideal classifier point was calculated. The point cut by the line where the Euclidean distance is minimized is the one offering TPR-FPR optimal balance. The optimum point is highlighted in red in Fig. 4B, and it corresponds to a threshold of *A* = 0.022 on the curve derived from batch-size *N* = 70.

As part of the comparative analysis, the power cost and computational complexity of digitally extracting biomarkers from memristive states were examined. Complexity estimates for biomarker extraction methods from Table 1 were obtained by simulating digital circuits for each strategy on a commercial Xilinx Artix-7 field-programmable gate array (FPGA). Figure 4C illustrates the performance of each strategy in terms of dynamic power and energy consumption (refer to table S1 for additional metrics and fig. S10 digital circuit schematics). The results indicate that batch strategy results in lower dynamic power and energy consumption equal, respectively, to 0.53 nW and 80 pJ for the evaluation of 70 samples constituting a cycle for the biomarker extraction. Overall, the analysis led us to conclude that batch solution delivered optimal performance. This optimal state arose from a coexistence of high accuracy, lower footprint, lower power consumption compared to competing methods, and notably, the ability to reduce the processing sampling frequency from 500 to 7.15 Hz.

This final aspect differed remarkably from the other methods, as it evaluated only the initial and final resistance states to determine the change in resistance (Δ*R*). Consequently, the operating frequency required for the biomarker extraction circuit was substantially reduced to 7.15 Hz, matching the duration of a batch size, which is 140 ms.

The duration of a batch size influenced the detection latency of our system, as the classification is performed over values contained in each batch. Figure 4E displays the time interval from when an auditory cue is played to when our system flags the events as detected. This interval is evaluated across different batch sizes. Here, a larger batch size implied a potential increase in the waiting time before a detection is finalized. These results are benchmarked against alternative mainstream software processing strategies for both LFP and AP signals, which involved Savitzky-Golay filtering of the trace and applying a peak-detection function from the signal processing library scikit-learn. The latency results for these two approaches are presented in the right segment of Fig. 4D. Specifically, we observe that our detection method, for a batch-size *N* = 70, offered a detection latency comparable to the LFP algorithmic method but at a much lower processing cost. Concerning the APs detection, we note that processing this waveform led to longer latency, even when leveraging a substantial amount of computational resources due to the inherent longer neural latency presented in Fig. 2E. Overall, according to Fig. 4D, for low batch sizes, memristors practically match mainstream software-based peak detection algorithms in terms of latency between behavioral stimulation of the rat and actual detection flag. Specifically, we obtained a median latency of 710 ms for the memristor-based approach compared to 760 ms for the LFP peak detection software, where in both cases 450 ms is due to neural latency.

Figure 4E showcases the detection performance of our system, reporting sensitivity, specificity, and accuracy for each feature extraction strategy. The same technique used to calculate the optimum *A* constant for batch in Fig. 4B was applied to the other two techniques, maxdiff and maxdrop, as illustrated in figs. S7B and S8B. The threshold that optimizes the ROC curve is different between different approaches. The obtained thresholds were used to determine their detection metrics. The pronounced elbow shape of the ROC curves shown in Fig. 4A is reflected in the detection results presented here. The batch method outperformed the others, achieving 97.89% accuracy with a sensitivity and specificity respectively equal to 96.36 and 97.95 % . This level of performance is fairly typical, with examples of different devices operating on the same neural trace and examples of the same device operating on different neural probe channels given in figs. S11 and 12 and figs. S12 and S13. It can be observed that the strategy adopted to optimize the threshold parameter *A* provided a robust approach to implement a system where the goal is to balance TPR and FPR. If the priority is minimizing false detections at the expense of reducing the true detection rate, the angle of the straight line α can be adjusted to favor a lower FPR.

To conclude the analysis, we evaluated the energy per operation of the system. We divided the system into two main parts—memristor encoding of the neural trace and digital processing and classification— and assessed their power contributions separately. Starting with the memristor-related stage, two primary operations were identified: memristor programming and memristor readout, aligning with prior approaches such as in (*31*, *40*). In the programming phase, the energy required for a 70-pulse batch size was calculated as ${\left(2V\right)}^{2}\times 0.5\;\text{mS}\times 100\frac{\text{ns}}{\text{pulse}}\times 1\times 70\;\text{pulses}=14\;\text{nJ}$. This estimation assumed a scenario where 2 V was applied directly across the terminals of the memristor, presuming any resistive drops along the electrode pathways were compensated for. In our experiments with stand-alone memristors, the maximum series resistance observed was 812 ohm. When subjected to a 2-V stimulus, the devices typically transitioned into a LRS. For the devices used in the experiments, the resistance in such a state, in the worst-case scenario, fell within a few kilohms. Consequently, for the purpose of energy evaluation, conductance value was set at 0.5 mS. In the reading phase, considering that the readout was performed once for every 70 values, the energy required for a 70-pulse batch size is ${\left(0.2\right)}^{2}\times 20\;\mu s\times 600\frac{\mu s}{\text{pulse}}\times 1\times 1\;\text{pulse}=0.48\;\text{nJ}$. The total energy 14 nJ + 0.48 nJ = 14.48 nJ.

Memristor technology shows a large potential for resistive range improvement, where a 100 kilohm resistive state at 2 V can be feasibly obtained. If the necessary assumptions are made in the programming phase, then ${\left(2V\right)}^{2}\times 20\;\mu S\times 100\frac{\text{ns}}{\text{pulses}}\times 1\times 70\;\text{pulses}=0.56\;\text{nJ}.$ Similarly, assuming a device can be read within 100 ns, the equivalent calculation gives ${\left(0.2\right)}^{2}\times 20\;\mu S\times 100\frac{\text{ns}}{\text{pulse}}\times 1\times 1\;\text{pulse}=80\;\text{fJ}$ for a total energy of 0.56 nJ + 80 fJ ≈ 0.56 nJ.

The second part was constituted by three substages, specifically, digitalizing the memristor resistance readings, carrying out the feature extraction strategy, and conducting threshold-based classification. For each of these contributions, power was calculated, and energy-per operation was derived. To digitalize the memristor readouts, a successive approximation register analog-to-digital converter (ADC) from the literature (*42*) compatible with our system was considered, contributing to the power consumption by 17 pW. The power required for feature extraction and threshold-based classification was then calculated on a 20-nm node technology, and the two contributions accounted respectively for 0.0627 and 0.0582 nW (details in table S2). Summing up the power contributions of the entire system, we got 4 nW + 0.017 nW + 0.0627 nW + 0.0582 nW = 4.14 nW, from which we derived an overall energy per operation of 0.58 nJ (see table S3). A detailed methodology for the power and energy evaluation is provided in Supplementary Text.

## DISCUSSION

A key difference between LFPs and APs is the much more standardized shape of the latter. The consistency in the shape of APs is attributed to the uniform sequence of ion channels opening and closing in the cell membrane (*43*). In contrast, LFPs do not exhibit a distinct or canonical form, as they arise from the combined activity of multiple neurons in a local area and reflect the synchronized synaptic inputs (*44*). The increased variation in signal characteristics appears to be the main challenge to reliable LFP events detection. A typical example is variation in the peak voltage of an LFP event, which may mean that certain instances of the class present excessively low peak voltages and fail to trigger resistive switching in the memristive device. This phenomenon is dominant over the intrinsic variation of our memristive devices (variation in resistive switching magnitude under the same waveform stimulus and initial resistive state) (*45*). To demonstrate this, we have illustrated the relationship between LFP events maximum voltages and RRAM detection, showing that 100% detection accuracy is possible (fig. S16). Changing the gain and offset settings applied to the neural waveform before it is fed to the memristive device so that even the weakest LFP class instances are detected represents a potential solution, but care must be taken to ensure no catastrophic overvoltage occurs at the highest voltage instances. A mechanism for determining the optimal gain/offset settings for maximum LFP event capture reliability is a natural next step. Furthermore, in terms of device-to-device variation, our method is intrinsically compensated against variations in the absolute resistive state of the device and can, in principle, be applied to any analog RRAM technology. This robustness stems from the temporally differential reading used by our algorithm, which remains unaffected by the device’s absolute resistive state. The performance characteristics of memristive devices have been extensively explored in prior studies (*38*). These devices typically exhibit a normal distribution (*46*) of switching voltages, i.e., the amount of switching occurring given an initial state and pulsing parameters tends to be fairly consistent across cycles (*47*).

The distinct nature of APs, which are generated by individual neurons, and LFPs, which arise from the collective activity of groups of neurons, leads to fundamental differences in their signal properties: LFPs are continuous, whereas APs are discrete entities. It is crucial to understand that the longer delay observed in the neuron burst depicted in Fig. 2 (C and D) does not necessarily mean that other neurons will exhibit similar delays relative to the LFP trace. However, the necessity of capturing the right neuron renders APs less suitable for the task. Even after identifying the correct neuron, its presence the following day cannot be guaranteed due to the inherent instability of the signal, typically caused by probe displacement in awake-animal settings.

In the original work (*40*) processing spiking activity, nonvolatile devices would saturate because of the unipolar nature of spikes. However, because LFPs have strong bipolar components, we observe that the resistive state of the memristor will travel in both directions when processing a typical waveform. Nevertheless, LFP traces cannot in general be guaranteed to offer bipolar peaks (that jut out of the noise floor and allow their exploitation via appropriate gain/offset settings), raising the possibility of resistive state saturation. How this is addressed will depend on the frequency of these “freak LFPs” and the importance of reliable channel operation. Given that the shape of LFPs, in general, depends on the specific biophysical and electrophysiological properties of the recorded network and the site of recording with respect to neuronal compartments (*48*), unipolar signals must be taken into account. An on-demand emergency reset feature might prove to be a good compromise. This mechanism would act as a safeguard, activating only when specific conditions indicate imminent saturation, thus remaining largely low power and potentially capable of being shared/multiplexed between multiple channels. We note that, in contrast, AP signals tend to be less forgiving and require counter measures against resistive state saturation. However, we also note that due to their shorter temporal dynamics they are also more amenable to the use of volatile (i.e., “self-resetting”) devices to counteract the problem (*49*). The practical behavior of volatile devices in the context of LFPs is an interesting path for further investigation.

We note that the extensive duration of LFPs (*13*) introduces a marked difference in how the MIS should be operated versus AP-oriented MIS systems. In AP-oriented systems, the memristor readout sampling rate is such that a (small) number of spikes fits within each sampling period [e.g., sampling window of 16.4 ms for APs lasting ≈1.5 ms in (*40*)], thus treating each AP as a discrete event that must be detected. In contrast, in LFP-oriented MIS systems despite operating at 500 Hz, the sampling period is smaller than the temporal extent of typical LFPs. As a result, each LFP is more likely to be detected within at least one sampling period. Understanding to what extent the readout sampling rate can be further reduced (along with the associated costs of data after processing and transmission off-implant) while maintaining sufficiently accurate detection is a key question to consider and will depend on required system accuracy and power specifications. The optimization of the timing dynamics for memristor programming, reading, and post-processing led to achieve a final classification frequency of 7.15 Hz, which directly affects power dissipation. The power required is not directly affected by the input, and different levels of neural activity will not change the number, type, and timing of operations carried out, but the cost of analog operations may increase with higher switching activity caused by input data. For instance, a high voltage on the resistive random access memory (RRAM) device increases energy consumption. Notably, the power consumption of the system is almost entirely programming and reading the memristor, which reduces the post-processing cost to sub-nanoWatt figures.

Our system performs comparably to system (*31*) that uses a memristor conductance modulation approach for epilepsy seizure detection, showing a power dissipation of 60.81 nW per channel versus our 4.14 nW per channel. This similarity primarily arises because both systems execute a similar number of memristor read and write operations per waveform, although ours operates on a single device, unlike their array of devices. Factors such as resistive state, biasing voltage range, pulse width, and sampling frequency dictate the final power dissipation. However, our system may have a natural advantage in power efficiency due to its simpler, smaller circuit that arise from carrying out all operations on a single device—at the expense of some waveform class discrimination capability (using a single integral value versus a 16-element vector). In addition, we compared our work with a threshold-based analog spike-detection implementation fabricated in 65-nm complementary metal-oxide semiconductor (CMOS) technology (*50*). As per equation *6* in (*50*), power consumption was Pt : 40 nW = 29.4 + 0.077 ∙ *f*, where *f* as the firing rate was set to 380. Setting *f* = 5, to account for lower frequency of oscillation of LFPs, we obtain an estimate of *P* = 29.8 nW. By comparison, our power consumption is lower by almost a factor of 7.

To put this work further into context, state-of-the-art spike-sorting solution shows 97.7% accuracy at 1.78 μW per channel in a 22-nm fully depleted silicon on insulator implementation, without linking to behavioral state detection (*51*). In a behavioral context, epilepsy seizure detection in 180 nm achieves 97.8% accuracy on intracortical activity using on-chip Ridge regression classification requiring 156.24 μW per channel (*8*). Last, advanced digital thresholding spike detection yields an accuracy of ≈96% at 280 nW per channel in 180 nm, and 38 nW per channel in 65 nm using Neuropixel intracortical data (*52*).

In our study, we have demonstrated single-trial LFP-based detection of behaviorally relevant signals, with high-performance accuracy of approximately 98%, thus leveraging a simple yet effective threshold system akin to those used in advanced commercial epilepsy monitoring systems (*10*). We achieved this outcome at a preliminary estimated power budget of about 0.1 μW for our devices’ technology, which could be dropped down to 4.14 nW for appropriately engineered, higher resistance devices. The proposed system efficiently and accurately interprets neural activity encoding reward conditioning memorization, triggered by sound. Its success with artificial cues suggests enhanced performance in detecting physiological patterns linked to clinical conditions.

We note that our work presents a memristor-based system for improving performance in the back-end of the neural waveform signal processing chain. However, in an end-to-end neural recording system, the front-end stage, involving waveform conditioning, also significantly affects the overall performance, including power dissipation. In this study, the signal-to-noise ratio of the waveform from the biological setup was <10 dB, with the raw LFPs around 80 μV. Therefore, the pre-amplifier specifications for bringing them to the magnitudes that can successfully induce resistive switching (~1 to 2 V) are similar to existing front-end circuits (~10,000 gain at the bandwidth relevant for capturing LFPs) (*53*). However, because these pre-amplified signals are applied directly to the memristive device, we obviate the need for ADC conversion, anti-aliasing filters etc., potentially enhancing the performance of the front-end part and the back-end. This aligns with emerging approaches linking neural activity to behavior using threshold crossing features (*17*, *54*), which do not require precise preservation of neural waveform shapes.

A natural next step will be to integrate the system by incorporating all components onto a single chip. Thanks to the compatibility of memristors with conventional CMOS, the monolithic integration of our system will enhance scalability and efficiency (*55*). For example, Reynolds *et al.* (*56*) presents an on-silicon flexible system that leverages the RRAM properties to implement a reconfigurable signal processor. In addition, a key question will be a detailed study of the power efficiency of this method as the number of channels increases, especially since any fully monolithic implementation is envisaged to include time-shared circuits. Our system’s lower-power performance would help with the objective of upscaling the number of channels without the risk of chronic heating. This prevents local damage and encapsulation of the heating source (*57*), which can compromise electrical connectivity.

The combination of competitive engineering performance with the temporal stability and clinical relevance of LFP signals suggest that LFP-oriented MIS is a promising approach for future applications in “coarse-grain” electrophysiology for research, bioprostheses, patient monitoring, and adaptive neuromodulation, e.g., by deep brain stimulation in Parkinson’s disease or essential tremor.

## MATERIALS AND METHODS

### Neural dataset acquisition

Before experiments, the animals underwent a surgical procedure during which 16 microwires (35 μm, isonel-coated tungsten; California Fine Wire Company) that were arranged in 4 × 4 arrays were lowered into the brain and fixed in position using dental cement (*35*). Neuronal activity was recorded from a freely moving animal at Bar-Ilan University. All procedures were approved by the Bar-Ilan University Institutional Animal Care and Use Committee; the ethics approval number is 02-01-2021. Extracellular recordings data were acquired, specifically within the VTA. During the experiment, an auditory cue was played to the animal. This cue always preceded the delivery of a reward and is supposed to generate an alteration in the VTA neuronal activity. This associative mechanism is obtained during an initial conditioning stage. Specifically, the VTA is well known for regulating reward consumption, learning, memory, and addiction behaviors through mediating dopamine release in downstream regions (*36*). The activity of channel 29 was chosen as it was relevant to our detection objectives. The activity was amplified, band-pass–filtered at 0.5 to 8000 Hz, and continuously sampled at 40 kHz using an OmniPlex data acquisition system (Plexon Inc).

### Memristor fabrication

The experiments were performed using solid-state titania/alumina-based devices with a vertical stack structure (from bottom to top): Pt/TiO_2 − *x*_/AlO*_x_*/Pt fabricated on SiO_2_/Si substrates using reactive magnetron sputtering in Ar/O_2_ ambient (for the dielectrics) and electron-beam evaporation (for the metals). Depending on the choice of materials, the devices can operate in either binary or in a smoother analog fashion as per (*38*). For the sake of this experiment, the analog nonvolatile behavior was chosen. Device readout is set at 200 mV, which is below their switching threshold (*38*).

### Custom board for memristor programming and reading

The memristors used in this experiment were programmed and measured using a parallel source-measure unit system (*37*). To facilitate this, a modified version of the instrument’s daughterboard was designed that included multiplexers, allowing devices in the memristor array to be individually selected to be exposed to the signal source. This daughterboard was then further modified to add signal processing modules, in this case a low pass filter for extracting the LFP from the recorded neural signal.

#### Signal processing

The neural signal conditioning consisted of a sixth order Butterworth filter implemented with a Sallen-Key topology, characterized by a cutoff frequency of 130 Hz. An adjustable gain stage completed the chain, allowing the gain to be fine-tuned from 1 up to a factor of 10 through a potentiometer. Figure S2 presents more details of the daughterboard processing stage.

#### Memristor programming and reading

SubMiniature version A connectors were mounted on the daughterboard to allow direct stimulation of the top electrode of a memristive device with the neural activity acquired from the rat. Moreover, switches were added to control the toggling between external source stimulation and the readout performed by the main board.

The operation sequence initiated by the switches involves a specific duration, wherein the memristor is connected to the LFP signal, followed by a phase dedicated to reading. During the time that the memristor is being programmed with the LFP, the main board’s circuitry concurrently conducts a high-impedance voltage measurement to record the signal applied to the memristor, ensuring the synchronization of the stimulus with the memristor’s response for later analysis.

Regarding the memristor state’s readout phase, the switches disconnect the device’s top electrode from the external signal source. The system then sets a readout voltage, in this case, 200 mV, and measures the current through a transimpedance amplifier connected to the memristor’s bottom electrode. The programming phase lasted for 10 μs, while the current readout to measure the resistance of the device required 600 μs. Between these operations, a dead time was assigned for the switches to toggle and stabilize, typically requiring a few hundred microsecond. The total time is adjusted to accommodate a complete programming and readout cycle at the desired frequency, which was set to 500 Hz in our experiment. For batch method implementation, the readout phase is performed only once for every 70 pulses. As a result, the device is mostly just programmed at a frequency of 500 Hz.

### Detection performance metrics

Event windows in the LFP waveforms were extracted algorithmically as the portion of the signal that starts from half the height of the first prominent peak following an auditory cue to the half height of the last peak. A repeatable and automated criterion for event window extraction was established and manually checked for the duration of the experiment. A detected event is defined as a flagging generated by the digital circuitry at the end of the batch, meaning the threshold conditioning was crossed by the examined Δ*R*. On the basis of that, the binary classification performance of the system is defined with the following:

1) True positives (TPs): There is at least one detected event falling in an event window.

2) False positives (FPs): All detected events fall outside an event window.

3) False negatives (FNs): There is no detected event in an event window.

4) True negatives (TNs): All batches occurring outside of an event window, excluding any false positives.

From these, we derived the following

$$\text{TPR}=\frac{\text{TP}}{\text{TP}+\text{FN}}$$

$$\text{FPR}=\frac{\text{FP}}{\text{FP}+\text{TN}}$$

Key performance metrics based on these terms were provided in Results:

1) Sensitivity: Ability of a model to correctly identify positive instances, calculated as $\frac{\text{TP}}{\text{TP}+\text{FN}}$.

2) Specificity: Ability of a model to correctly identify negative instances, calculated as $\frac{\text{TN}}{\text{TN}+\text{FP}}$.

3) Accuracy: Overall correctness of the model’s predictions, calculated as $\frac{\text{TP}+\text{TN}}{\text{TP}+\text{TN}+\text{FP}+\text{FN}}$.

### Data annotation and validation

The data used for our experiment were manually labeled, leveraging automated peak extraction algorithms for comparison with ground truth. This detailed labeling and validation help ensure the accuracy and reliability of the detection algorithms tested.

## Notes

**Correction (12 December 2024):** The funding source “RAEng Chair in Emerging Technologies (Grant No. CiET1819/2/93)” was mistakenly omitted from the funding statement, and this error was only recently brought to the attention of the Editorial Office. The funding source has been added to the funding statement. The full text and PDF versions of the paper have been updated.

## Supplementary Materials

### The PDF file includes:

Supplementary Text Figs. S1 to S16 Tables S1 to S4 Legend for movie S1 References
