# Supplementary material for: Single-trial detection of auditory cues from the rat brain using memristors
Source: Sci Adv. 2024 Sep 4;10(36):eadp7613. doi: 10.1126/sciadv.adp7613 (PMC11373585; doi:10.1126/sciadv.adp7613)
Supplement: Supplementary file 1 — Supplementary Text Figs. S1 to S16 Tables S1 to S4 Legend for movie S1 References [file sciadv.adp7613_sm.pdf]

Science Advances
AAAS

Supplementary Materials for

Single-trial detection of auditory cues from the rat brain using memristors

Caterina Sbandati *et al.*

Corresponding author: Caterina Sbandati, caterina.sbandati@ed.ac.uk

Sci. Adv. **10**, eadp7613 (2024)
DOI: 10.1126/sciadv.adp7613

The PDF file includes:

Supplementary Text
Figs. S1 to S16
Tables S1 to S4
Legend for movie S1
References

Other Supplementary Material for this manuscript includes the following:

Movie S1

Supplementary Text

Power evaluation methodology

To evaluate the total power, we split the system into four blocks, as depicted in **fig. S15:** memristor-encoding stage, read-outs digitalization, feature-extraction and classification.

1) The contribution of the memristive stage, constituted by programming and reading of the device, is $4\ nW$.

2) The contribution of read-outs digitalization was estimated from the work *(42)*, selected from the Murmann ADC 2024 survey *(59)*. The selected work implements a fully-differential charge redistribution successive approximation register (SAR) analog to digital converter (ADC) manufactured in 65 nm technology and operating at a sampling frequency of the same order of magnitude as us. The power performance of the ADC from **Fig. 20** in *(42)* was adapted relying on Walden Figure or Merit (FoM): $244\ fJ = \frac{P}{2^{ENOB} \times f_s}$, where $ENOB = 8.3$ and $f_s = 10\ Hz$. In our case, we need a 5-bit ADC as this precision is assumed in subsequent processing steps to correctly differentiate resistance changes across the functional resistive range of the device (38). Assuming the same 2.66 LSB-error implied by the ENOB of the original, we get an $ENOB = 3.3$. If we run such an ADC at a frequency $f_s = 7.15\ Hz$, we obtain a power from the Walden FoM of $P = 17\ pW$.

3) For the feature extraction three methods were investigated: *maxdiff*, *maxdrop* and *batch.* We implemented these methods on a Xilinx Artix-7 xc7a100tcsg324-1 FPGA, by using the AMD Vivado 2022.2 tool. Resources utilization, dynamic power and energy estimation are collected in **table S1**. According to the results, *Batch* method emerged as the optimal feature extraction strategy. Afterwards, the architecture modelling the *batch* method was synthesized on ASIC to better estimate the performance of a silicon implementation. The synthesis was run using CMOS 180 nm process @ 1.8 V from TSMC on the Cadence Genus tool. Results (area, power, energy) are collected in **table S2**. Moreover, to facilitate comparison with state-of-the-art architectures, the results were scaled targeting bulk 65 nm @ 1.1 V and multi-gate 20 nm @ 0.9 V, based on the scaling practice (58). The feature extraction in $20\ nm$ technology accounts for $62.7\ pW$.

4) To conclude, the classification stage was also synthesized on ASIC following the same procedure as for the *batch* method. When scaled to the 20 nm process, the classification stage contributed $58.2\ pW$ in terms of power.

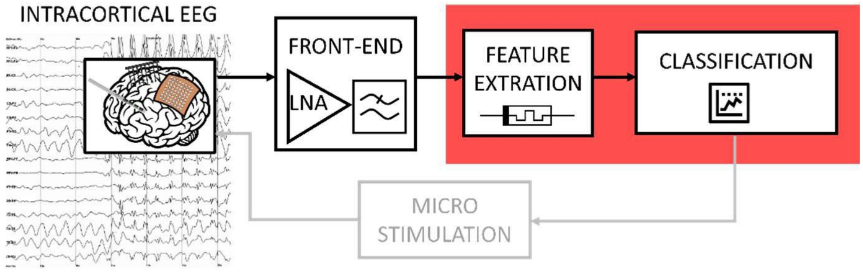

Fig. S1. Block diagram of a typical brain-machine interface system. The neural signal is acquired by a front-end stage, typically comprising a gain stage and a filter. Subsequently, features are extracted and used to classify the neural activity. The two steps highlighted in red in the figure, feature extraction and classification, are the focus of this article, specifically aiming to implement an accurate and yet efficient solution.

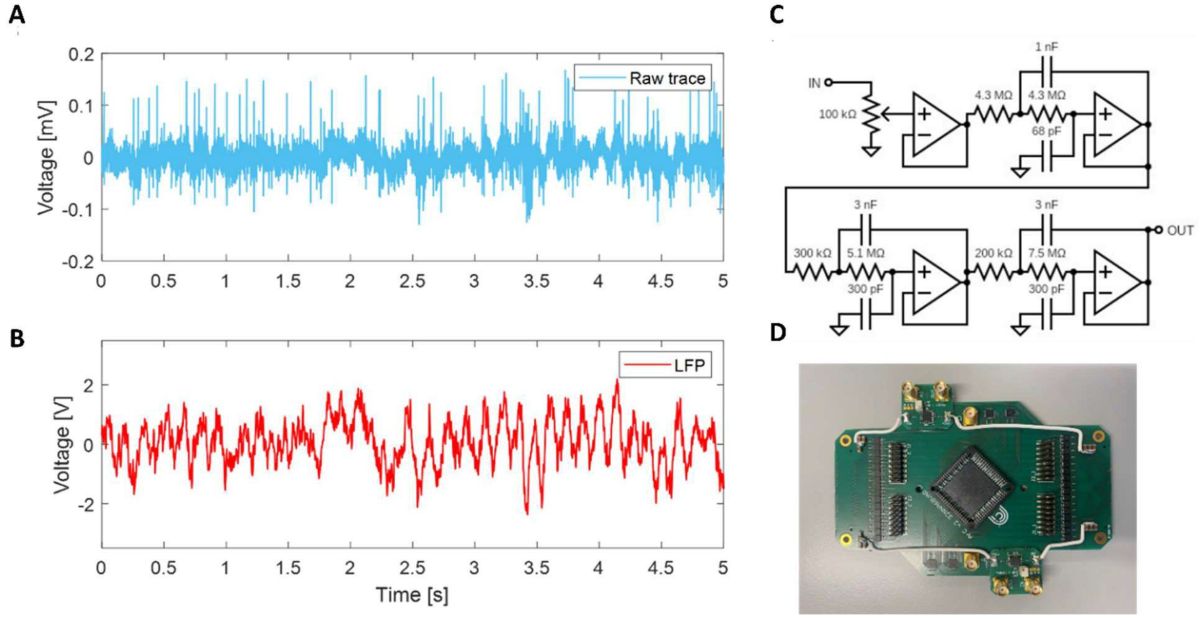

Fig. S2. **Overview of LFP front-end signal processing and experimental protocol**. A) Raw extracellular activity from channel WB29. (B) Local Field Potential signal. (C) Schematic of the circuit used to extract LFP from raw activity. It consists of a variable gain amplifier and a 6th order low-pass Butterworth filter implemented with a Sallen-Key topology featuring a cut-off frequency of 130 Hz. (D) Photo of the daughterboard used to apply the LFP signal to a memristive device. It hosts both the front-end circuit and the memristive devices.

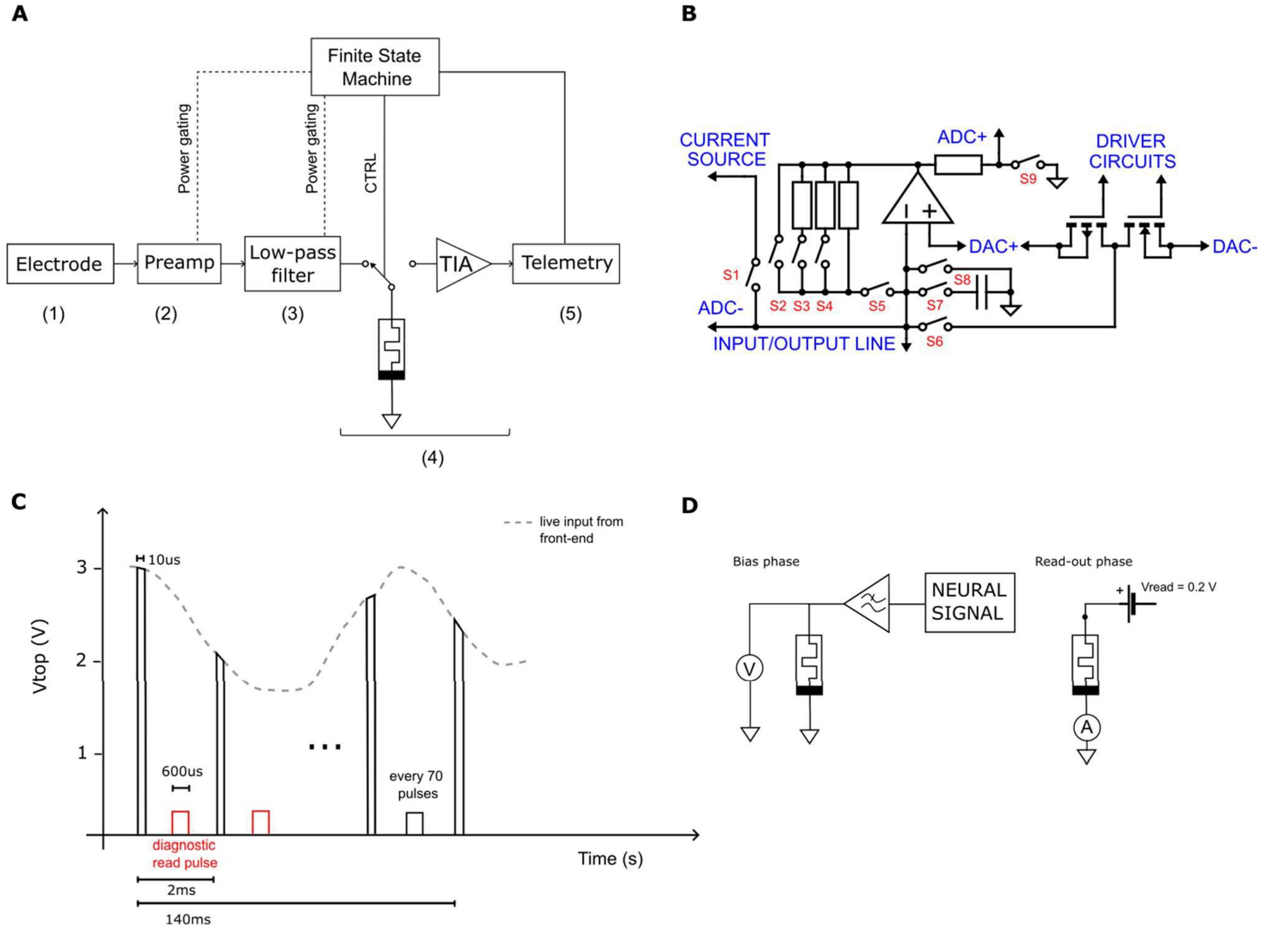

Fig. S3: System and timing details. (A) Schematic of the envisioned system *in operation*. The neural signal is acquired by the electrode (1), amplified (2) and a low-pass filtered (3). The memristor is alternatively connected to either the *live feed*, or connected to the transimpedance amplifier (TIA) (4). Telemetry (5) processes the memristor resistive readings. The ArC TWO board acting as finite state machine powers the amplifier and active filter, and orchestrates the alternation (CTRL) of the two configurations in (4). (B) Channel topology schematic of the ArC TWO instrument used in the experiment. If ADC+ is not grounded, the ADC reading measures the current into the channel, if grounded, the ADC reading measures the voltage at the channel. Adapted from *(37),* (2020) Springer-Nature CC-BY. (C) Timing diagram showing the voltage applied to the top electrode of the memristor. During the *bias phase*, the neural signal is applied to the memristor for $\mathbf{10}\ \boldsymbol{\mu s}$, marked as solid line overlapping the live input. During the *read-out phase*, a $\mathbf{0.2}\ \boldsymbol{V}$ sub-threshold voltage is applied to read the device without altering its current state. A $\mathbf{2}\ \boldsymbol{ms}$ period includes delay time between phases. Telemetry uses readings every 70 samples for processing; additional read-outs (in red) enhance monitoring and will not occur in real applications. (D) Circuit system diagram depicting the memristor switching between two configurations. In *bias phase* it connects to an external LFP, with voltage monitored at the top terminal. In *read-out phase* it is connects to a $\mathbf{0.2}\ \boldsymbol{V}$ reading voltage, with a TIA read the current through the device.

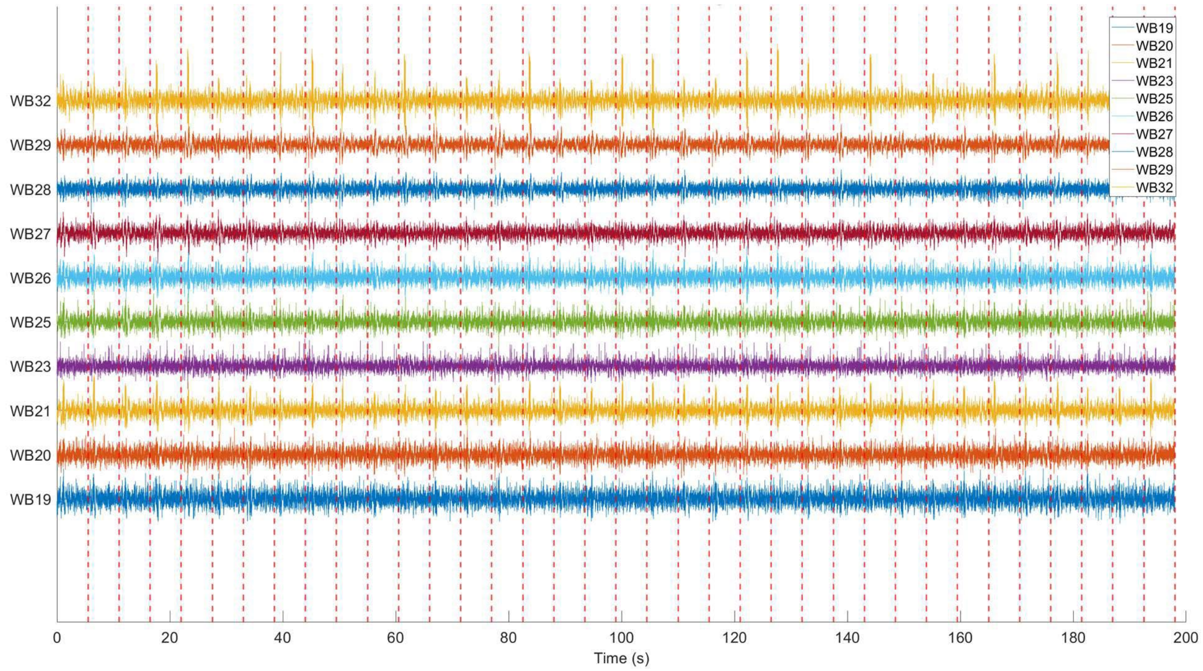

Fig. S4. Segment of the Local Field Potential recording obtained from 9 microwires implanted in the rat's brain. Dashed red lines mark instants when the auditory cue was presented to the animal. Following these cues, a discernible pattern emerges in the collective neural activity. The specific channel utilized in this experiment is identified as channel WB29.

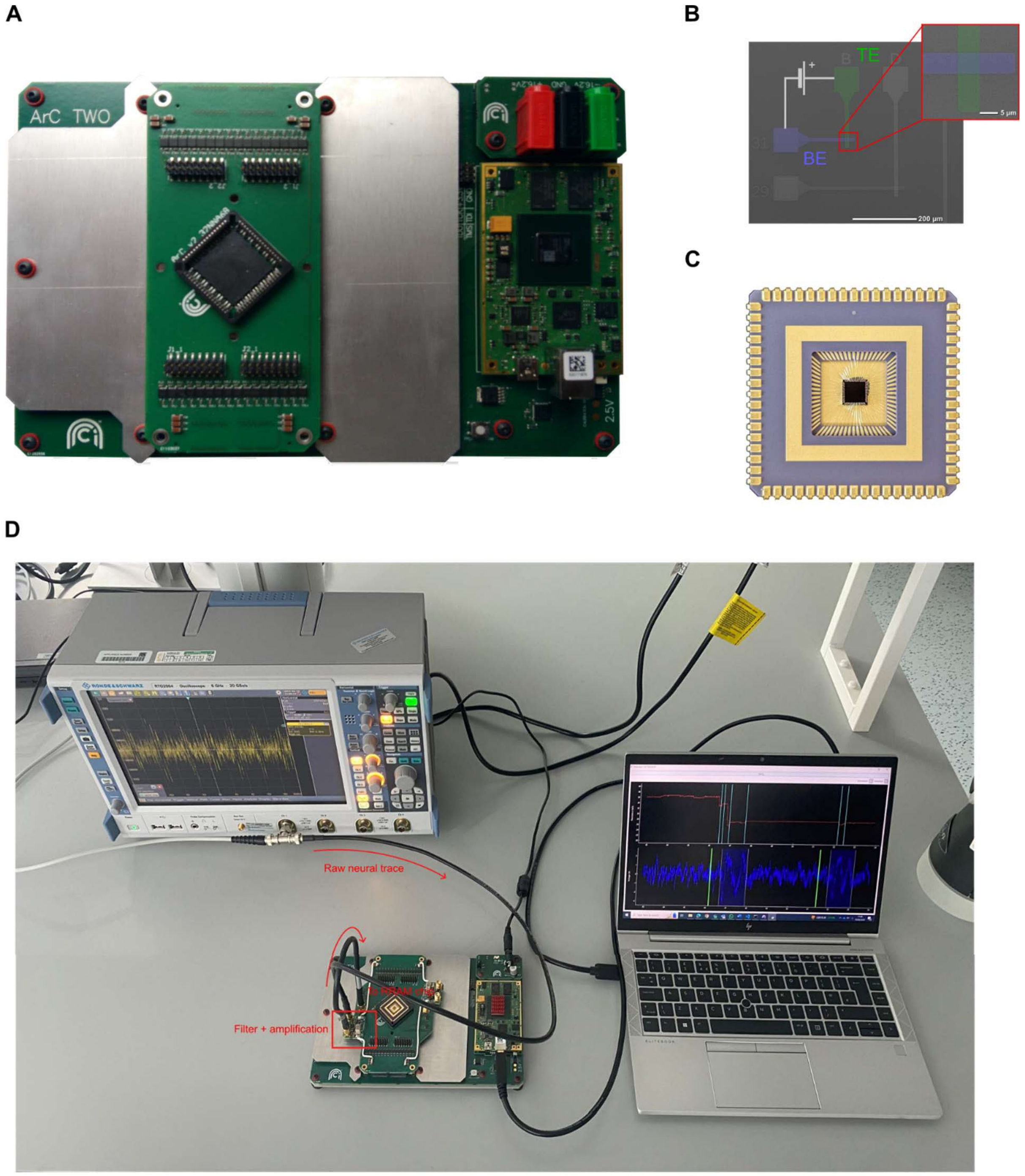

Fig. S5. Overview of custom hardware ArC TWO and experimental set-up [40]. (A) Picture of fully assembled system PCBs, including base board, device-under-test interfacing daughterboard, FPGA development board and power supply board (B) Scanning electron microscope (SEM) image of stand-alone memristors. The top electrode (TE) of a device is highlighted in green and the bottom electrode (BE) in blue; the area of their intersection identifies a memristive device, with a zoomed-in view of the area shown. (C) Memristive array packaged in a PLCC68 package.
(D) Experimental set-up: the arrows indicate the flow of the signal. The neural signal is generated with an arbitrary signal generator, passed to the filter and amplification stage and then directed to the memristive chip.

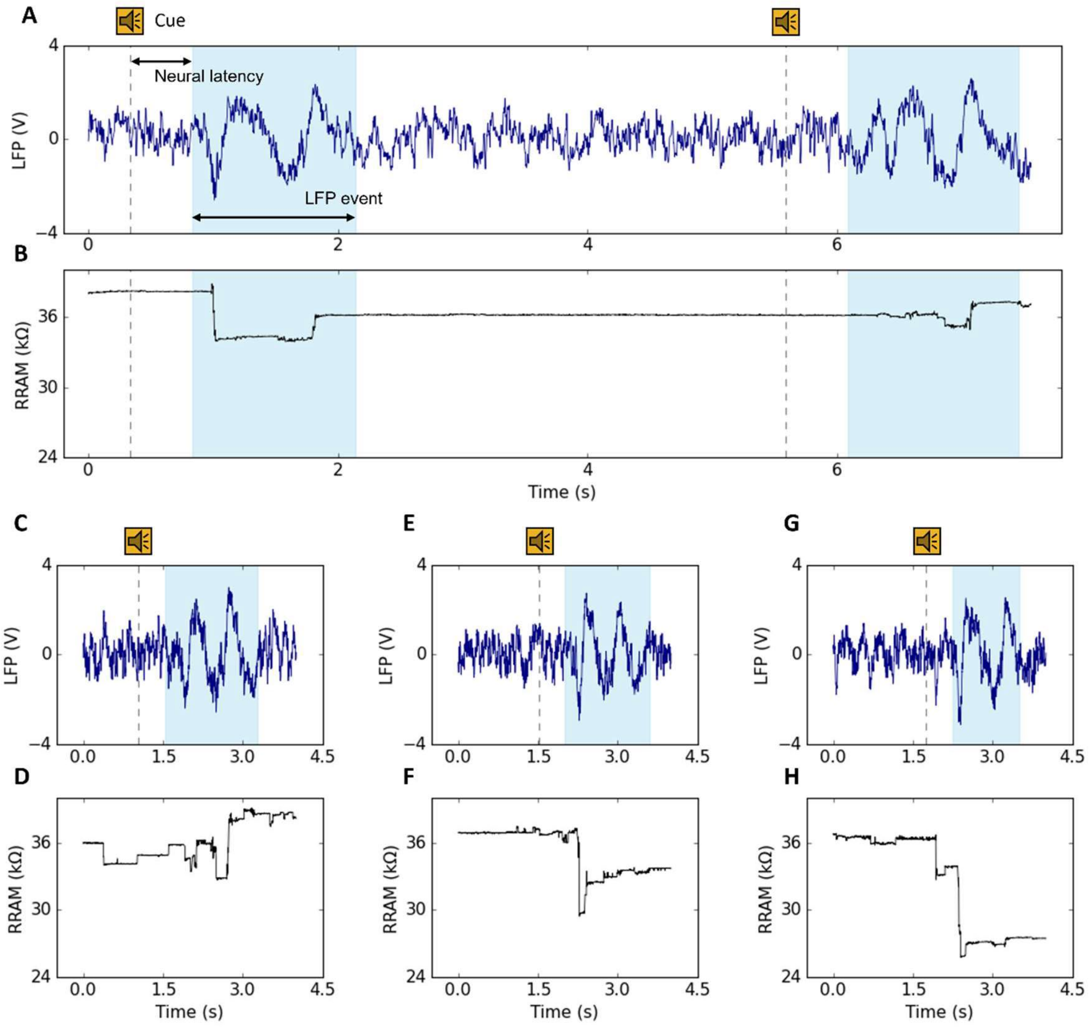

Fig. S6. Biasing of a memristor device with Local Field Potential to extract auditory cue patterns. (A) the amplified Local Field Potential signal biasing the RRAM device, and (B) the resistive state of the RRAM device. The green dashed lines mark moments when auditory cues were presented to the rat. (C-H) Zoom-ins of the experiment showcasing details of the memristive encoding of LFP events. allowing for the identification of neural activity responses triggered by the auditory cues.

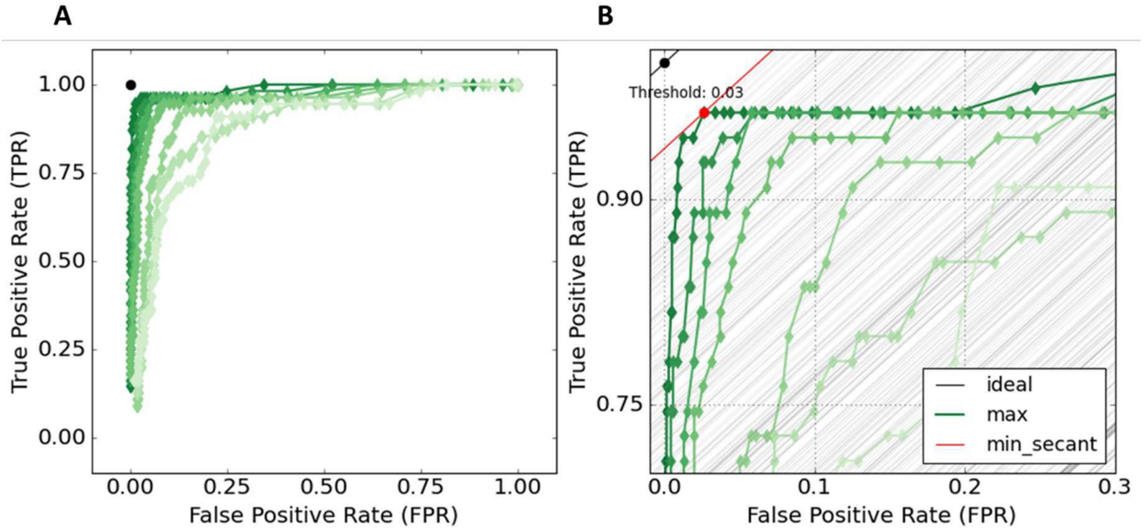

Fig. S7: Receiver Operating Characteristic for "maxdrop" detection strategy. (A) Different batch sizes ranging from 70 to 490. (B) Optimization process to extract the optimum threshold value using the min distant tangent method. Here, the optimized threshold setting is 0.03.

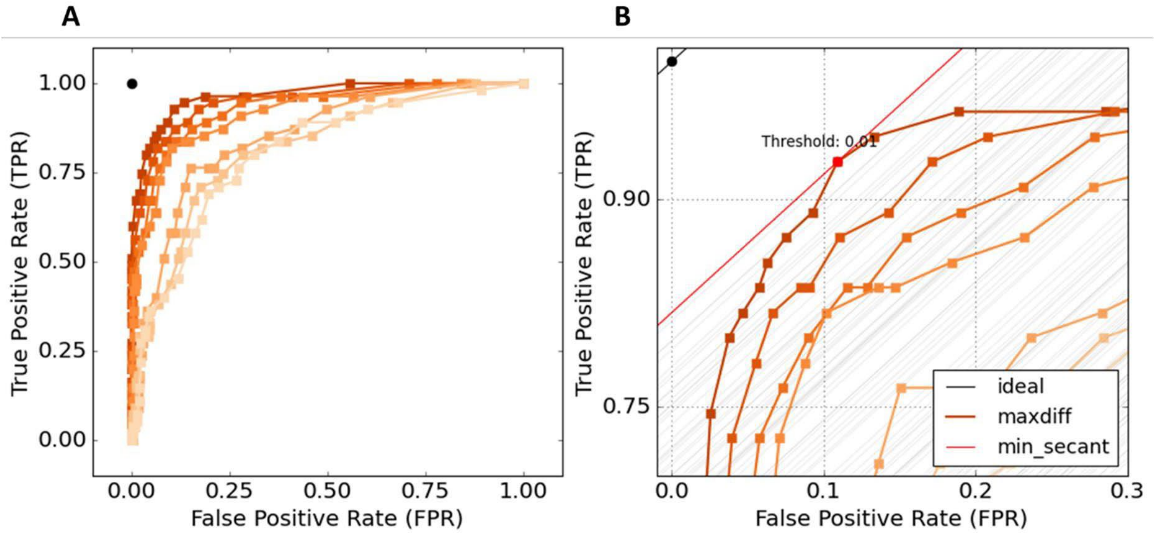

Fig. S8. Receiver Operating Characteristic for "maxdiff" detection strategy. (A) Different batch sizes ranging from 70 to 490. (B) Optimization process to extract the optimum threshold value using the min distance secant method. Here, the optimum threshold value was identified as 0.01.

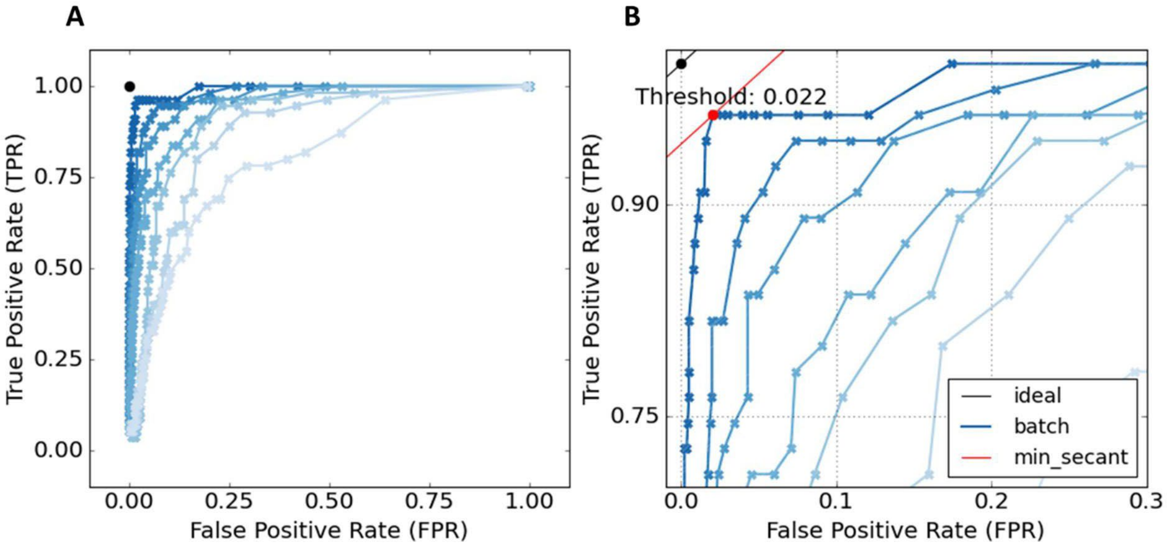

Fig. S9. Receiver Operating Characteristic for "batch" detection strategy. (A) Different batch sizes ranging from 70 to 490. (B) Optimization process to extract the optimum threshold value using the min distance secant method. Here, the optimum threshold value was identified as 0.022.

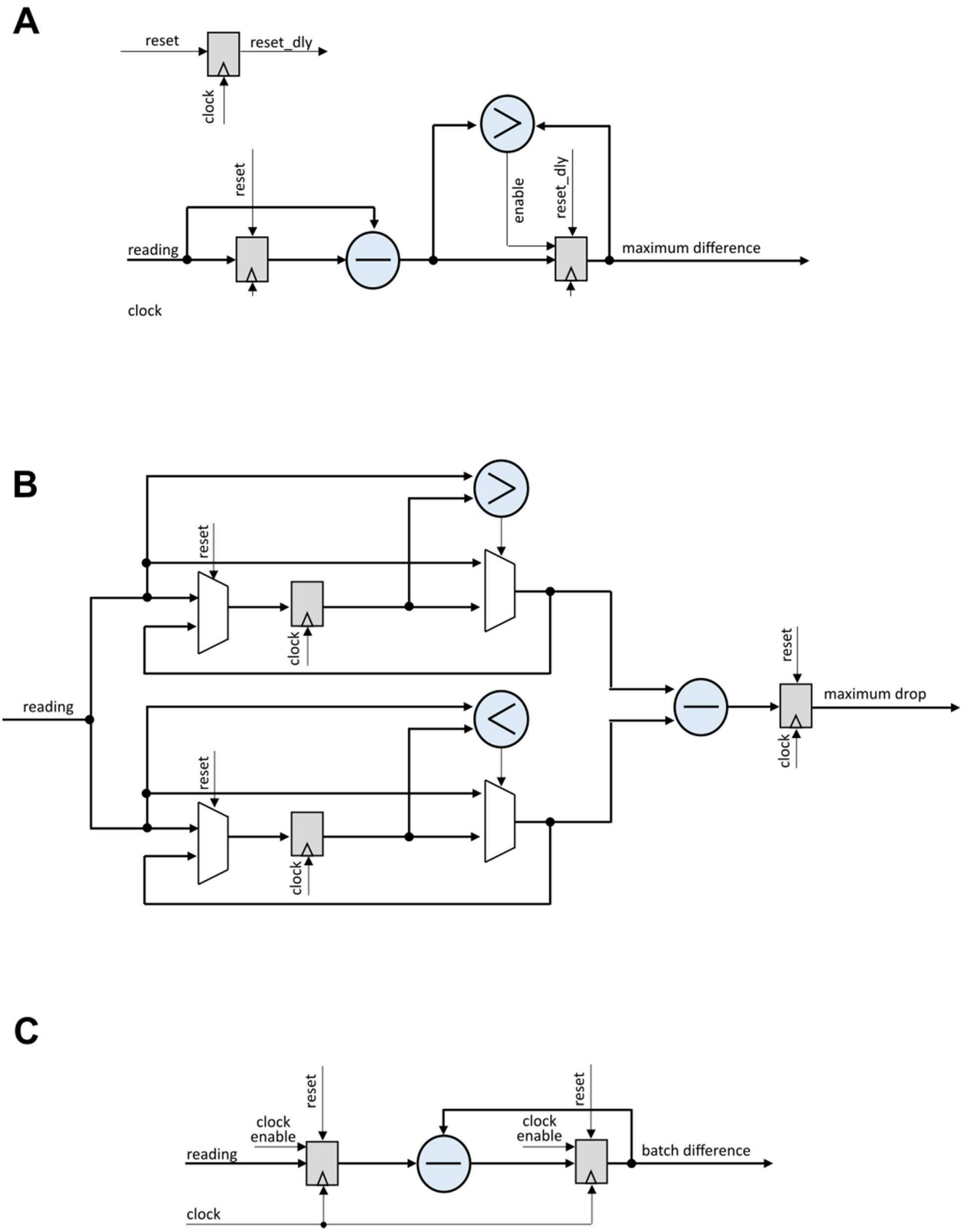

Fig. S10. Circuit schematics of the FPGA-based threshold strategies. (A) maxdiff, (B) maxdrop, and (C) batch.

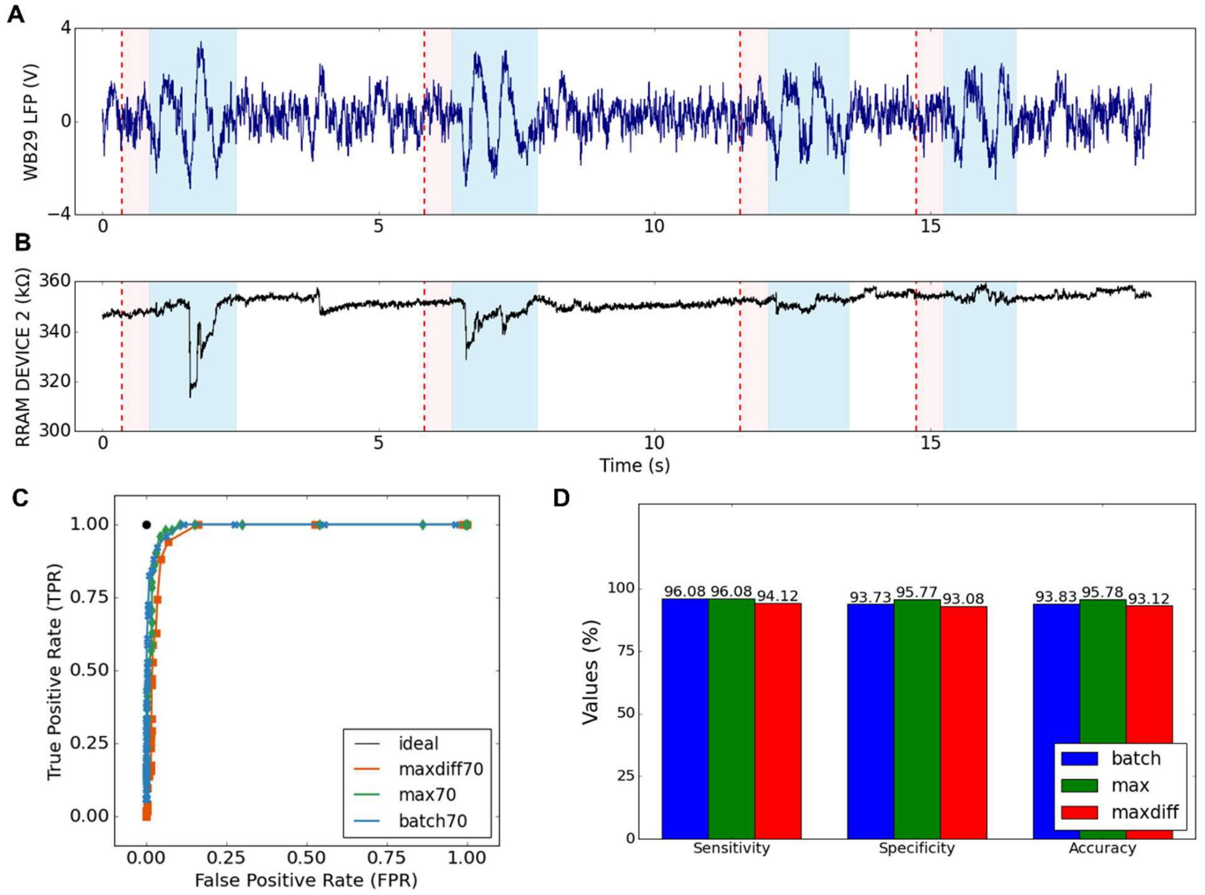

Fig. S11. Device variability analysis example 1. (A) Channel WB29 LFP signal and (B) corresponding memristive resistance state. Notably, the selected memristor presents a higher resistance range, characterized by low resistive range of about $\boldsymbol{100\ k\Omega}$. This higher resistance would directly contribute to lower power consumption during memristor programming and reading phases. (C) ROC curves of the three methods: "maxdiff", "maxdrop" and "batch". (D) Performance metrics setting the threshold at $\boldsymbol{A = 0.008}$. Detection success is not influenced by memristor resistive range but by resistance changes, ensuring consistent performance and potential power savings irrespective of device variability.

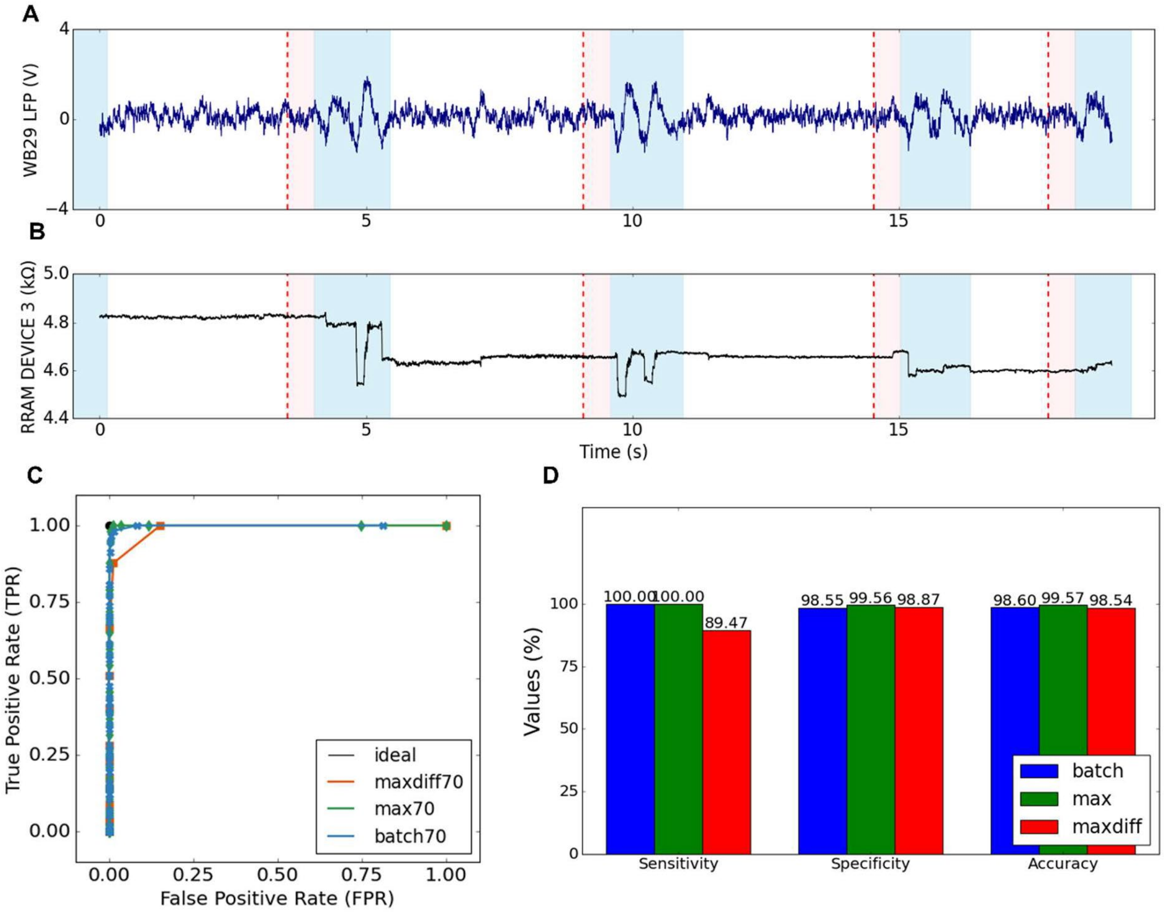

Fig. S12. Device variability analysis example 2. (A) Channel WB29 LFP signal and (B) corresponding memristive resistance state. This time the device exhibits a very small resistance operating range. (C) ROC curve of the three methods: "maxdiff", "maxdrop" and "batch". (D) Detection metrics.

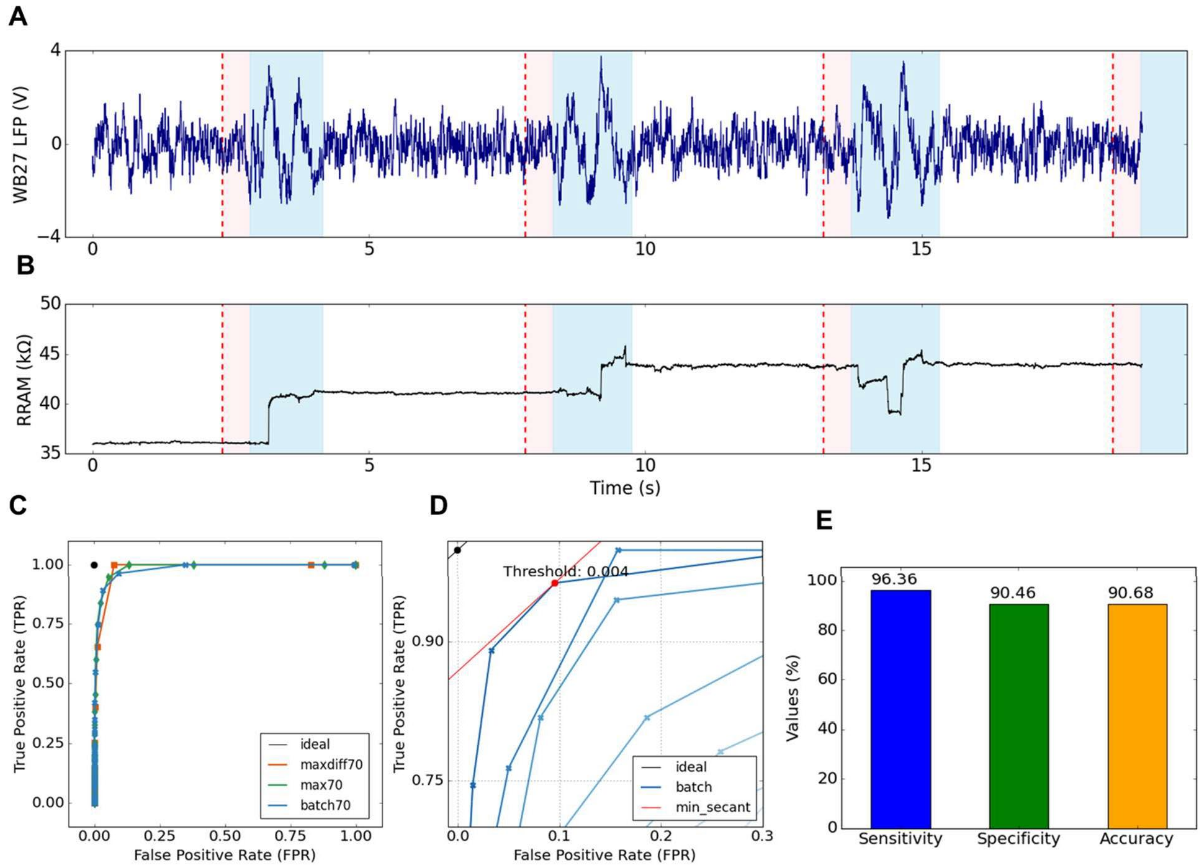

Fig. S13. Channel variability analysis, example 1. (A) Channel WB27 was input to a memristive device, (B) and the resistive response of the device was monitored. We can observe the signal was similar to the initial channel WB27, and the resistance successfully performs encoding of the LFP event class within the light blue windows. (C) ROC curves using “maxdiff”, “maxdrop” and “batch”. (D) Optimization process to select an optimum threshold, in this case equal to $\boldsymbol{A} = \mathbf{0.004}$. (E) Detection metrics for the “batch” method.

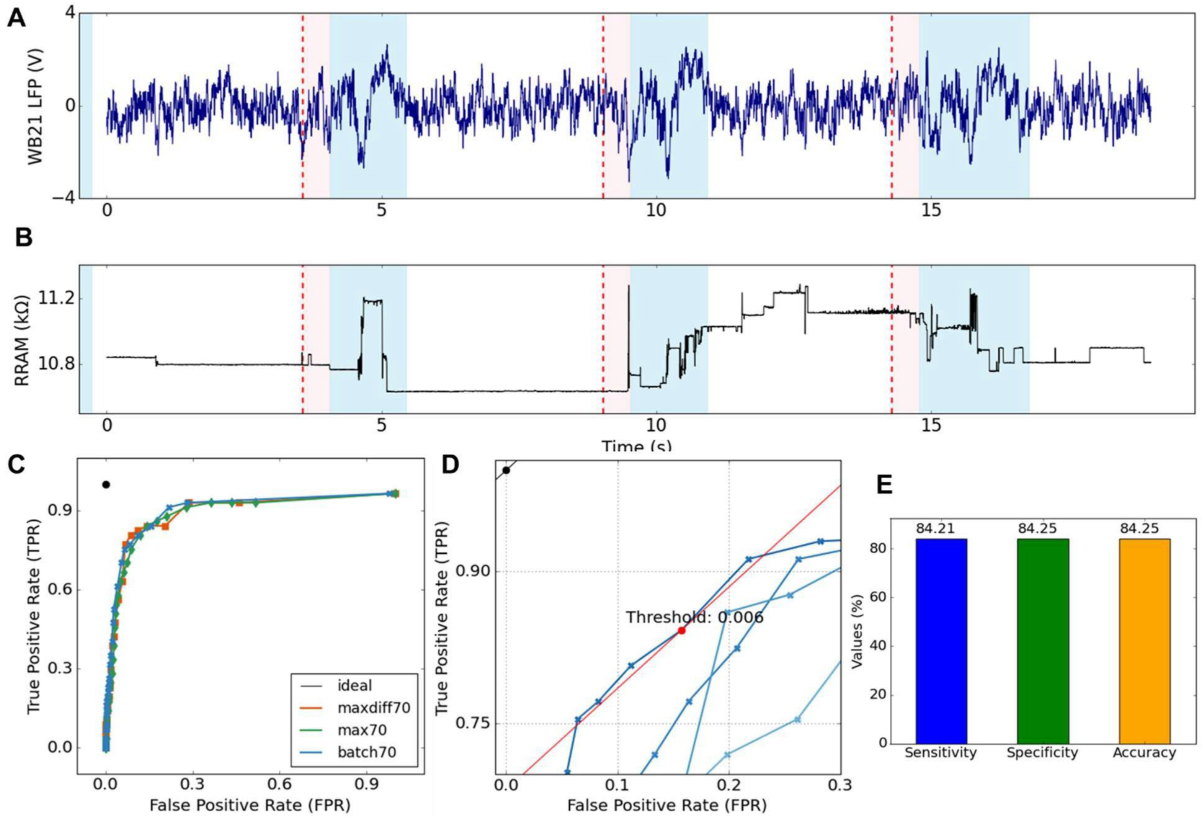

Fig. S14. Channel variability analysis, example 2. (A) Channel WB21 was input to a memristive device, (B) and the resistive response of the device was monitored. Channel WB21 exhibited peaks that were more difficult to detect, and this led to a noisier shape of the memristive device response. (C) ROC curves using “maxdiff”, “maxdrop” and “batch”. (D) Optimization process to select an optimum threshold, in this case equal to A=0.004. (E) Detection metrics for the "batch" method show decreased performance due to the less distinct LFP signal input to the memristive device.

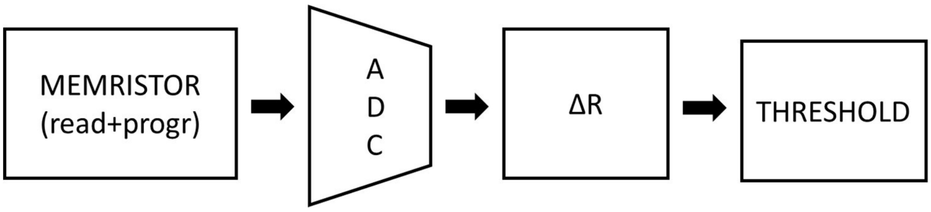

Fig. S15. System splitting into blocks for power and energy evaluation.

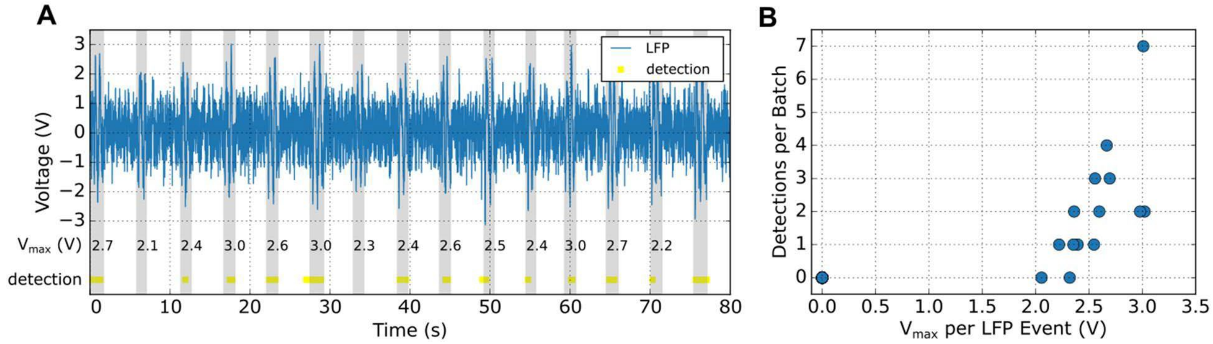

Fig. S16. Analysis of LFP Voltage Peaks and RRAM Detection Correlation. (A) Extract of LFP data highlighting instances where detections are missed, particularly during low voltage peak events. The yellow markers indicate individual detection points, while the numerical labels beneath the panel represent the maximum voltage ($\boldsymbol{V_{max}}$) for each corresponding event. (B) A scatter plot demonstrating the positive correlation between the maximum voltage of LFP Events and the number of detections per batch, suggesting higher detection rates with increased voltage levels.

	maxdiff	*maxdrop*	*batch*
LUTs	7	21	4
FFs	11	15	10
Dyn. Power [nW]	0.94	1.26	0.53
Simulation Time [ms]	150	150	150
Energy [nJ]	0.14	0.19	0.08

Table S1. Hardware Resource Utilization and Power Estimation for FPGA Implementation. Detailed summary of the hardware resource utilization, dynamic power and energy estimation for the FPGA-based implementation of our system. Utilizing the Xilinx Artix-7 xc7a100tcsg324-1 and Vivado 2022.2 tool, we retrieved the consumption of Look Up Tables (LUTs) and Flip Flops (FFs). Dynamic power was estimated through post-implementation timing simulations, which involved generating a Switching Activity Interchange Format (SAIF) file. This file reflects the activity of internal nodes to ensure an accurate dynamic power estimation. Finally, we estimated the energy per operation considering a batch of N=70 samples, with resistance values quantized as 5-bit integer data. The clock frequency was set to 500 Hz.

	Batch Feature extraction + Classification
Area (180 nm) [μm^2]	2289.31
Power [μW] (180 nm) @ 100MHz	378
Power [nW] (180 nm) @ 500Hz	1.89
Simulation Time [s]	0.15
Energy [μJ] @ 100 MHz	56.7
Energy [nJ] @ 500 Hz	0.284

Area (65 nm) [μm^2]	183.1448
Power [μW] (65 nm) @ 100MHz	99.4
Power [nW] (65 nm) @ 500 Hz	0.497
Simulation Time [s]	0.15
Energy [μJ] @ 100 MHz	14.9
Energy [nJ] @ 500Hz	0.0746

Area (20 nm) [μm^2]	25.18241
Power [μW] (20 nm) @ 100MHz	24.2
Power [nW] (20 nm) @ 500 Hz	0.121
Simulation Time [s]	0.15
Energy [μJ] @ 100 MHz	3.63
Energy [nJ] @ 500Hz	0.0181

Table S2. Area and Power Estimation for ASIC Synthesis: Detailed summary of area, dynamic power, and energy estimation for the ASIC-based synthesis of our system. Cadence Genus tool was used on an ASIC 180 nm process at 1.8 V, and the values were scaled respectively targeting Multi-Gate HP 20nm at 0.9V and Bulk 65nm at 1.1V, using (57).

	Memristor	*ADC*	*Feature extraction (Batch)*	*Classification (Threshold)*	***Total***
Power [nW]	4	0.017	0.0627	0.0582	4.138
Energy [nJ]	0.56	0.002	0.0094	0.00873	0.580

Table S3. Power and Energy-per-operation evaluation for blocks depicted in Fig. S14. Post-processing and thresholding present the values for 20nm ASIC implementation.

	Gupta et al. (31)	*Liu et al. (30)*	*Liu et al. (33)*	*This work*
Accuracy [%]	76.9	93.46	95.00	97.89
Power [nW]	300/channel	1.39×10^{3} /class	60.81/channel	4.14/channel
Classifications per second	63	10	1	8
Energy per operation [nJ]	0.105	140	60.81	0.58
Number of channels	NA	1	16	1
Memristor stack	Ti/Pt/TiOx/Pt	TiN/TaOx/HfOy/TiN	TiN/TaOx/HfOy/TiN	Ti/Pt/TiOx/Pt

Table S4. Table of comparison for neural-signal processing studies involving memristors state of the art. Here, channel refers to the number of neural recording channels used in the system. For (33) the power and energy were evaluated at a sampling frequency of 10kHz, to enable comparison with state-of-the-art CMOS ASIC.

Movie S1. Real-time detection of memory tasks

Online auditory cue detection based on memristive integrating sensor system. The lower blue trace represents local field potentials recorded with implanted microwire in the ventral tegmental area of an awake freely moving rat. The upper trace is the memristive response to the local field potential applied to it. Green lines denote the presence of an auditory cue, also played in the background of the video. Cyan lines indicate the corresponding detections that follow. The LFP regions of interest are also highlighted.

REFERENCES AND NOTES

1. J. R. Wolpaw, J. D. R. Millán, N. F. Ramsey, Brain-computer interfaces: Definitions and principles. *Handb. Clin. Neurol.* **168**, 15–23 (2020).

2. C. N. Heck, D. King-Stephens, A. D. Massey, D. R. Nair, B. C. Jobst, G. L. Barkley, V. Salanova, A. J. Cole, M. C. Smith, R. P. Gwinn, C. Skidmore, P. C. Van Ness, G. K. Bergey, Y. D. Park, I. Miller, E. Geller, P. A. Rutecki, R. Zimmerman, D. C. Spencer, A. Goldman, J. C. Edwards, J. W. Leiphart, R. E. Wharen, J. Fessler, N. B. Fountain, G. A. Worrell, R. E. Gross, S. Eisenschenk, R. B. Duckrow, L. J. Hirsch, C. Bazil, C. A. O'Donovan, F. T. Sun, T. A. Courtney, C. G. Seale, M. J. Morrell, Two-year seizure reduction in adults with medically intractable partial onset epilepsy treated with responsive neurostimulation: Final results of the RNS System Pivotal trial. *Epilepsia* **55**, 432–441 (2014).

3. R. A. Andersen, T. Aflalo, S. Kellis, From thought to action: The brain-machine interface in posterior parietal cortex. *Proc. Natl. Acad. Sci. U.S.A.* **116**, 26274–26279 (2019).

4. A. B. Rapeaux, T. G. Constandinou, Implantable brain machine interfaces: first-in-human studies, technology challenges and trends. *Curr. Opin. Biotechnol* **72**, 102–111 (2021).

5. M. Shoaran, B. A. Haghi, M. Taghavi, M. Farivar, A. Emami-Neyestanak, Energy-efficient classification for resource-constrained biomedical applications. *IEEE J. Emerg. Sel. Top Circuits Syst.* **8**, 693–707 (2018).

6. N. Even-Chen, D. G. Muratore, S. D. Stavisky, L. R. Hochberg, J. M. Henderson, B. Murmann, K. V. Shenoy, Power-saving design opportunities for wireless intracortical brain–computer interfaces. *Nat. Biomed. Eng.* **4**, 984–996 (2020).

7. K. Gadhoumi, J. M. Lina, F. Mormann, J. Gotman, Seizure prediction for therapeutic devices: A review. *J. Neurosci. Methods* **15**, 270–282 (2016)

8. C. H. Cheng, P. Y. Tsai, T. Y. Yang, W. H. Cheng, T. Y. Yen, Z. Luo, X. H. Qian, Z. X. Chen, T. H. Lin, W. H. Chen, W. M. Chen, S. F. Liang, F. Z. Shaw, C. S. Chang, Y. L. Hsin, C. Y. Lee, M. D. Ker, C. Y. Wu, A fully integrated 16-channel closed-loop neural-prosthetic CMOS SoC

with wireless power and bidirectional data telemetry for real-time efficient human epileptic seizure control. *IEEE J. Solid-State Circuits* **53**, 3314–3326 (2018).

9. M. Shoaran, U. Shin, M. Shaeri, Intelligent neural interfaces: An emerging era in neurotechnology, in *2024 IEEE Custom Integrated Circuits Conference (CICC)* (IEEE, 2024), pp. 1–7.

10. H. Kassiri, A. Chemparathy, M. T. Salam, R. Boyce, A. Adamantidis, R. Genov, Electronic sleep stage classifiers: A survey and VLSI design methodology, *IEEE Trans. Biomed. Circuits. Syst.* **11**, 177–188 (2016)

11. L. Drew, Decoding the business of brain–computer interfaces, *Nat. Electr.* **6**, 90–95 (2023)

12. S. Waldert, Invasive vs. non-invasive neuronal signals for brain-machine interfaces: Will one prevail?, *Front Neurosci.* **10**, 295 (2016).

13. G. Buzsáki, C. A. Anastassiou, C. Koch, The origin of extracellular fields and currents-EEG, ECoG, LFP and spikes, *Nat. Rev. Neurosci.* **13**, 407–420 (2012)

14. E. R. Kandel, J. D. Koester, S. H. Mack, S. A. Siegelbaum, *Principle of Neural Science* (McGraw-Hill, ed. 5, 2013).

15. T. Zhang, M. Rahimi Azghadi, C. Lammie, A. Amirsoleimani, R. Genov, Spike sorting algorithms and their efficient hardware implementation: A comprehensive survey, *J. Neural Eng.* **20**, 021001 (2023).

16. M. Tambaro, M. Bisio, M. Maschietto, A. Leparulo, S. Vassanelli, fpga design integration of a 32-microelectrodes low-latency spike detector in a commercial system for intracortical recordings. *Dent. Dig.* **1**, 34–53 (2021).

17. E. M. Trautmann, S. D. Stavisky, S. Lahiri, K. C. Ames, M. T. Kaufman, D. J. O’Shea, S. Vyas, X. Sun, S. I. Ryu, S. Ganguli, K. V. Shenoy, Accurate Estimation of Neural Population Dynamics without Spike Sorting. *Neuron* **103**, 292–308.e4 (2019).

18. A. Destexhe, J. Goldberg, *Encyclopedia of Computational Neuroscience* (Springer, 2015).

19. S. R. John, W. Dagash, A. N. Mohapatra, S. Netser, S. Wagner, Distinct dynamics of theta and gamma rhythmicity during social interaction suggest differential mode of action in the medial amygdala of sprague dawley rats and C57BL/6J mice. *Neuroscience* **493**, 69–80 (2022).

20. Y. Baumel, D. Cohen, State-dependent entrainment of cerebellar nuclear neurons to the local field potential during voluntary movements. *J. Neurophysiol.* **126**, 112–122 (2021).

21. A. Jackson, T. M. Hall, Decoding local field potentials for neural interfaces, *IEEE Trans. Neural Syst. Rehabil. Eng.* **25**, 1705–1714 (2017)

22. S. D. Stavisky, J. C. Kao, P. Nuyujukian, S. I. Ryu, K. V. Shenoy, A high performing brain-machine interface driven by low-frequency local field potentials alone and together with spikes. *J. Neural. Eng.* **12**, 036009 (2015).

23. D. Gervasoni, S. C. Lin, S. Ribeiro, E. S. Soares, J. Pantoja, M. A. L. Nicolelis, Global forebrain dynamics predict rat behavioral states and their transitions. *J. Neurosci.* **24**, 11137–11147 (2004).

24. E. Covi, E. Donati, X. Liang, D. Kappel, H. Heidari, M. Payvand, W. Wang, Adaptive extreme edge computing for wearable devices. *Front Neurosci.* **15**, 611300 (2021).

25. M. A. Zidan, J. P. Strachan, W. D. Lu, The future of electronics based on memristive systems. *Nat. Electron.* **1**, 22–29 (2018).

26. R. Waser, M. Aono, Nanoionics-based resistive switching memories. *Nat. Mat.* **6**, 833–840 (2007)

27. I. Tzouvadaki, P. Gkoupidenis, S. Vassanelli, S. Wang, T. Prodromakis, Interfacing biology and electronics with memristive materials. *Adv Mater.* **35,** 2210035 (2023).

28. A. Serb, A. Corna, R. George, A. Khiat, F. Rocchi, M. Reato, M. Maschietto, C. Mayr, G. Indiveri, S. Vassanelli, T. Prodromakis, Memristive synapses connect brain and silicon spiking neurons. *Sci. Rep.* **10**, 2590 (2020).

29. C. Dias, D. Castro, M. Aroso, J. Ventura, P. Aguiar, Memristor-based neuromodulation device for real-time monitoring and adaptive control of neuronal populations. *ACS Appl. Electron. Mater.* **4**, 2380–2387 (2022).

30. I. Gupta, A. Serb, A. Khiat, T. Prodromakis, Improving detection accuracy of memristor-based bio-signal sensing platform. *IEEE Trans. Biomed. Circuits Syst.* **11**, 203–211 (2017).

31. Z. Liu, J. Tang, X. Li, P. Yao, Y. Lin, D. Liu, B. Hong, H. Qian, H. Wu, Multichannel parallel processing of neural signals in memristor arrays. *Sci. Adv* **6,** eabc4797 (2020).

32. Z. Liu, J. Tang, B. Gao, P. Yao, X. Li, D. Liu, Y. Zhou, H. Qian, B. Hong, H. Wu, Neural signal analysis with memristor arrays towards high-efficiency brain–machine interfaces. *Nat. Commun* **11**, 4234 (2020).

33. J. Van Assche, M. F. Carlino, M. D. Alea, S. Massaioli, G. Gielen, "From sensor to inference: end-to-end chip design for wearable and implantable biomedical applications" in *BioCAS 2023–2023 IEEE Biomedical Circuits and Systems Conference, Conference Proceedings* (IEEE, 2023), pp. 1–5.

34. M. Sharifshazileh, K. Burelo, J. Sarnthein, G. Indiveri, An electronic neuromorphic system for real-time detection of high frequency oscillations (HFO) in intracranial EEG. *Nat. Commun.* **12**, 3095 (2021).

35. N. D. Peer, H. G. Yamin, D. Cohen, Multidimensional encoding of movement and contextual variables by rat globus pallidus neurons during a novel environment exposure task. *iScience* **25**, 105024 (2022).

36. J. Cai, Q. Tong, Anatomy and function of ventral tegmental area glutamate neurons. *Front Neural Circuits* **16**, 867053 (2022).

37. P. Foster, J. Huang, A. Serb, S. Stathopoulos, C. Papavassiliou, T. Prodromakis, An FPGA-based system for generalised electron devices testing. *Sci. Rep.* **12**, 13912 (2022).

38. S. Stathopoulos, A. Khiat, M. Trapatseli, S. Cortese, A. Serb, I. Valov, T. Prodromakis, Multibit memory operation of metal-oxide Bi-layer memristors. *Sci. Rep.* **7**, 17532 (2017).

39. L. Michalas, A. Khiat, S. Stathopoulos, T. Prodromakis, Electrical characteristics of interfacial barriers at metal - TiO2 contacts. *J. Phys. D Appl. Phys.* **51**, 425101 (2018).

40. I. Gupta, A. Serb, A. Khiat, R. Zeitler, S. Vassanelli, T. Prodromakis, Real-time encoding and compression of neuronal spikes by metal-oxide memristors. *Nat. Commun.* **7**, 12805 (2016).

41. F. S. Nahm, Receiver operating characteristic curve: overview and practical use for clinicians. *Korean J. Anesthesiol.* **75**, 25–36 (2022).

42. H. Wang, X. Wang, A. Barfidokht, J. Park, J. Wang, P. P. Mercier, A battery-powered wireless ion sensing system consuming 5.5 nW of average power. *IEEE J. Solid-State Circuits* **53**, 2043–2053 (2018).

43. A. L. Hodgkin, A. F. Huxley, A quantitative description of membrane current and its application to conduction and excitation in nerve. *J. Physiol.* **117**, 500–544 (1952).

44. O. Herreras, Local field potentials: Myths and misunderstandings. *Front. Neural Circuits* **10**, 10.3389/fncir.2016.00101 (2016).

45. I. Gupta, A. Serb, A. Khiat, M. Trapatseli, T. Prodromakis, Spike sorting using non-volatile metal-oxide memristors. *Faraday Discuss.* **213**, 511–520 (2019).

46. A. G. Panca, A. Serb, S. Stathopoulos, S. K. Garlapati, T. Prodromakis, Automated RRAM measurements using a semi-Automated probe station and ArC ONE interface, in *IEEE International Conference on Microelectronic Test Structures* (IEEE, 2023), pp. 1–4.

47. S. Stathopoulos, A. Serb, A. Khiat, M. Ogorzałek, T. Prodromakis, A memristive switching uncertainty model. *IEEE Trans. Electron. Devices* **66**, 2946–2953 (2019).

48. G. T. Einevoll, C. Kayser, N. K. Logothetis, S. Panzeri, Modelling and analysis of local field potentials for studying the function of cortical circuits, *Nat. Rev. Neurosci.* **14**, 770–785 (2013)

49. I. Gupta, A. Serb, A. Khiat, R. Zeitler, S. Vassanelli, T. Prodromakis, Sub 100 nW volatile nano-metal-oxide memristor as synaptic-like encoder of neuronal spikes. *IEEE Trans. Biomed. Circuits Syst.* **12**, 351–359 (2018).

50. E. Yao, Y. Chen, A. Basu, A 0.7 V, 40 nW compact, current-mode neural spike detector in 65 nm CMOS. *IEEE Trans. Biomed. Circuits Syst.* **10**, 309–318 (2016).

51. Y. Chen, B. Tacca, Y. Chen, D. Biswas, G. Gielen, F. Catthoor, M. Verhelst, C. Mora Lopez, An online-spike-sorting IC using unsupervised geometry-aware OSort clustering for efficient embedded neural-signal processing. *IEEE J. Solid-State Circuits* **58**, 2990–3002 (2023).

52. Z. Zhang, P. Feng, A. Oprea, T. G. Constandinou, Calibration-free and hardware-efficient neural spike detection for brain machine interfaces. *IEEE Trans. Biomed. Circuits Syst.* **17**, 725–740 (2023).

53. R. R. Harrison, C. Charles, A low-power low-noise CMOS for amplifier neural recording applications. *IEEE J. Solid-State Circuits* **38**, 958–965 (2003).

54. F. R. Willett, D. T. Avansino, L. R. Hochberg, J. M. Henderson, K. V. Shenoy, High-performance brain-to-text communication via handwriting. *Nature* **593**, 249–254 (2021).

55. X. Jiang, C. Sbandati, G. Reynolds, C. Wang, C. Papavassiliou, A. Serb, T. Prodromakis, S. Wang, A neural recording system with 16 reconfigurable front-end channels and memristive processing/memory unit, in *21st IEEE Interregional NEWCAS Conference (NEWCAS)* (IEEE, 2023), pp. 1–5.

56. G. Reynolds, X. Jiang, A. Serb, T. Prodromakis, S. Wang, An Integrated CMOS/memristor bio-processor for re-configurable neural signal processing, in *2023 IEEE Biomedical Circuits and Systems Conference (BioCAS)* (IEEE, 2023), pp. 1–5.

57. H. Kambic, G. M. Saidel, Characterization of tissue morphology, angiogenesis, and temperature in the adaptive response of muscle tissue to chronic heating, *Lab. Invest.* **78**, 1553–1562 (1998)

58. A. Stillmaker, B. Baas, Scaling equations for the accurate prediction of CMOS device performance from 180 nm to 7 nm. *Dermatol. Int.* **58**, 74–81 (2017).

59. B. Murmann, ADC Performance Survey 1997–2024 (2024); https://github.com/bmurmann/ADC-survey.
